# Single-cell RNA sequencing and ATAC sequencing identify novel biomarkers for bicuspid aortic valve-associated thoracic aortic aneurysm

**DOI:** 10.3389/fcvm.2024.1265378

**Published:** 2024-04-08

**Authors:** Xu-Wen Liu, Pei Wang, Li Zhang, Yu Zhu, Jun-Yu Zhai, Chang-Nan Wang, Jun Li, Jian Xiao

**Affiliations:** ^1^ School of Medicine, Guangxi University, Nanning, China; ^2^ Department of Cardiothoracic Surgery, Changzheng Hospital, Naval Medical University, Shanghai, China; ^3^ School of Health Sciences and Engineering, University of Shanghai for Science and Technology, Shanghai, China; ^4^ Department of Cardiac Surgery, Zhongshan Hospital, Fudan University, Shanghai, China; ^5^ School of Life Sciences, Shanghai University, Shanghai, China

**Keywords:** bicuspid aortic valve, thoracic aortic aneurysm, scRNA-seq, ATAC-seq, diagnostic biomarkers, UMAP, WGCNA

## Abstract

**Introduction:**

Bicuspid aortic valve (BAV) is the most prevalent congenital cardiovascular defect and known to cause thoracic aortic aneurysms (TAAs). To improve our understanding of BAV pathogenesis, we characterized the cellular composition of BAV tissues and identified molecular changes in each cell population.

**Methods:**

Tissue samples from two patients with BAV and two heart transplant donors were analyzed using single-cell RNA sequencing, assay for transposase-accessible chromatin using sequencing, and weighted gene coexpression network analysis for differential gene analysis. TAA-related changes were evaluated by comparing the proportion of each cell type and gene expression profiles between TAA and control tissues. Further, by combining our single-cell RNA sequencing data with publicly available data from genome-wide association studies, we determined critical genes for BAV.

**Results:**

We found 20 cell subpopulations in TAA tissues, including multiple subtypes of smooth muscle cells, fibroblasts, macrophages, and T lymphocytes. This result suggested that these cells play multiple functional roles in BAV development. Several differentially expressed genes, including CD9, FHL1y, HSP90AA1, GAS6, PALLD, and ACTA2, were identified.

**Discussion:**

We believe that this comprehensive assessment of the cellular composition of TAA tissues and the insights into altered gene expression patterns can facilitate identification of novel diagnostic biomarkers and therapeutic targets for BAV-associated TAA.

## 1 Introduction

Bicuspid aortic valve (BAV), the most common congenital cardiovascular defect with an incidence of 1%–2% in the general population (1), can cause aortic disease and increase the risk of aortic-related diseases in affected patients (2–5). BAV is one of the causes of thoracic aortic aneurysms (TAAs) (6–10), which occur when the aortic blood vessel expands, causing the aorta to become >1.5 times the standard arterial diameter. Aortic aneurysms are typically asymptomatic during progressive enlargement until acute aortic dissection occurs, which is linked to a high mortality rate. TAA is a dangerous, potentially fatal, and asymptomatic condition that can involve one or more segments of the thoracic aorta (4, 6).

Recent studies have revealed the complex and unique pathogenesis of BAV-associated TAA, encompassing gene mutations, hemodynamics, mechanical stress, oxidation and inflammation, as well as their interactions (10). However, previous research has paid limited attention to cell-specific changes in the aortic wall of patients with BAV-associated TAA.

To address this gap, herein we investigated TAA tissues from patients with BAV using single-cell RNA sequencing (scRNA-seq) and assay for transposase-accessible chromatin using sequencing (ATAC-seq) to elucidate the molecular processes underlying BAV-associated TAA occurrence and development. scRNA-seq is a powerful tool to characterize gene expression in individual cells; moreover, it allows for the identification of intercellular variations and can reveal complex cell populations and regulatory relationships between genes. ATAC-seq is a method to assess open chromatin and evaluate genome-wide chromatin accessibility in individual cells. We also performed weighted gene coexpression network analysis (WGCNA), a method increasingly employed in disease and gene association analysis, to identify key genes and better comprehend disease regulatory mechanisms. We believe that our findings will facilitate the identification of novel diagnostic biomarkers and therapeutic targets for BAV-associated TAA and enhance our understanding of its pathogenesis.

## 2 Materials and methods

### 2.1 Tissue collection

Aortic aneurysm patient tissues were obtained from two patients with BAV, and control tissues were obtained from two heart transplant recipients. This study was approved by the Ethics Committee of Shanghai Changzheng Hospital, all participants were informed about our research (Table 1).

**Table 1:** Control group patient information form.

	Patient 1	Patient 2
Age	64	32
Sex	Male	Male
BMI	23.71	24.32
Primary cause	Myocardiopathy	Myocardiopathy
Risk factors		
Diabetes	No	No
Hypertension	Yes	Yes
Cerebrovascular disease	No	No
Previous history of cardiac surgery	No	No
Smoking	No	Yes
Drinking	Yes	Yes
Aortic valve condition	Normal	Normal
Aortic diameter	31 mm	34 mm
Left ventricular end diastolic diameter	59 mm	61
Pulmonary arterial pressure	48 mmHg	44 mmHg

In the control group, the aortic wall of patients undergoing heart transplantation was examined. During the surgical procedure of removing the donor heart, the aortic wall was excised and trimmed. The aortic valve of the recipient patient was tricuspid, and the aorta showed no significant dilation, with no other vascular lesions.

### 2.2 ATAC-seq

We lysed 50,000 cells in cold ATAC resuspension buffer containing 0.1% NP40, 0.1% Tween-20, and 0.01% digitonin, followed by incubation on ice for 3 min. Subsequently, 1 ml precooled resuspension buffer containing only 0.1% Tween-20 was added, mixed by inversion three times, and centrifuged at 500 ×g for 5 min at 4°C. The supernatant thus obtained was discarded; 50 μl transposition mix was then added, followed by incubation at 37°C on a rotating mixer at 1,000 RPM for 30 min. DNA fragments were purified using the Qiagen MinElute PCR Purification Kit and amplified by 5–9 PCR cycles. After library detection and quality control, sequencing was performed on the Illumina HiSeq/NextSeq platform. Raw reads obtained upon ATAC-seq were filtered to remove adapters and contaminants before being aligned to the reference genome. High-quality mapped reads (MPAQ ≥30) were used for subsequent analyses.

### 2.3 Dataset collection and processing

The following four gene expression profile datasets were retrieved from Gene Expression Omnibus (GEO) (http://www.ncbi.nlm.nih.gov/geo): GSE5180, GSE83675, GSE61128, and GSE2615 (11–13). In addition, we utilized self-test datasets, which included two aneurysm patients and two control groups. Single-cell data were also derived from GSE155468 (14).

The original dataset was subjected to background correction and normalization using the Affy R package. Datasets requiring data conversion were log_2_ transformed. In cases where multiple probes corresponded to the same gene, we calculated the average value to determine its expression level. After merging the five datasets, we used the Bioconductor sva package in R to eliminate batch effects, resulting in a batch effect-free matrix for subsequent analyses.

### 2.4 Weighted gene coexpression network construction

WGCNA is employed to analyze gene modules with high biological significance and investigate the relationship between gene networks and diseases. The WGCNA R package is used for constructing a weighted gene coexpression network. First, hierarchical clustering is performed to detect outliers and remove abnormal samples. A soft threshold value of *β* = 12 is selected based on *R*^2^ > 0.9 to achieve scale-free topology. The minimum number of module genes is then set to 25 and module merging threshold is set to 0.25 to construct a weighted coexpression network and identify gene modules. Finally, the correlation between modules and phenotypes is determined, and modules highly correlated with phenotypes are subjected to further analysis.

### 2.5 Chromatin accessibility analysis

The FastQC tool was applied for quality control of clean data after removing adapters and low-quality data. Clean data were aligned to the reference genome using Bowtie2. The ATACseqQC R package was used to create an insertion fragment length distribution map of the sample, which facilitated the preliminarily evaluation of the quality of the ATAC experiment. The deepTools multiBamSummary and plotCorrelation programs were employed for correlation analyses of duplicate samples. deepTools was also used to generate transcriptional start site (TSS) enrichment heatmaps, indicating the enrichment status of data on TSS and comparing the enrichment status of different samples in the TSS region. The alignment results were then processed using MACS2 for peak calling, with *q* < 0.05 set as the threshold. The ChIPseeker R package was utilized for annotation and visualization of peak calling data.

### 2.6 Gene ontology (GO) and Kyoto encyclopedia of genes and genomes (KEGG) pathway enrichment analyses

The GO system contains structured and computable information on gene and gene product functions. As a knowledge base, KEGG facilitates evaluating functions of genes and pertinent signaling pathways. We performed functional enrichment analysis using the clusterProfiler R package, and the results of the enrichment analysis were visualized using the ggplot2 R package. *p* < 0.05 indicated significant enrichment.

### 2.7 Single-cell data processing and cell subpopulation identification

We used the Seurat R package for data processing, ensuring that each gene was expressed in at least 3 cells, with each cell expressing at least 250 genes. Cells containing >5% mitochondrial genes were filtered out, and data were normalized using the log-normalization function. Subsequently, clustering based on differences in gene expression between cells was performed to analyze variations among different cell populations. To achieve this, highly variable genes were selected using the FindVariableFeatures function, and their expression matrix dimension was reduced using the RunUMAP function. The two-dimensional uniform manifold approximation and projection (UMAP) algorithm was applied using the RunUMAP function in Seurat to visualize cells. The FindAllMarkers function was employed to identify cell marker genes and cell subtypes, and the FindMarkers function was utilized to identify differentially expressed genes (DEGs).

### 2.8 Screening of potential diagnostic biomarkers

To identify novel and key biological markers for TAA, we employed three methods: single-cell differential gene analysis, ATAC differential gene analysis, and WGCNA. DEGs obtained on applying these methods were intersected and sorted by differential expression multiple. From this analysis, we chose the top six genes exhibiting the most significant differences as the final hub genes.

### 2.9 Pseudo-time analysis

To investigate changes in cell state, we utilized the Monocle2 R package. DEGs over the pseudo-time among cluster cell transitions were calculated with the differentialGeneTest function. DDRTree was employed for dimensionality reduction and visualization, while plot_cell_trajectory was utilized to plot the minimum spanning tree on cells. The SMC Contractile, SMC Proliferating, Fibroblast, and MSC clusters were partitioned and visualized using Monocle3, highlighting hub gene expression changes from the start to the end of the pseudo-time process.

### 2.10 Statistical analysis

GraphPad Prism was used for statistical analysis. An independent sample test was applied to assess the significance of the differences in hub gene expression among patients with BAV. Statistical significance was set at *p* < 0.05.

## 3 Results

### 3.1 Weighted gene coexpression network construction and module preservation analysis

**Figure 1:**
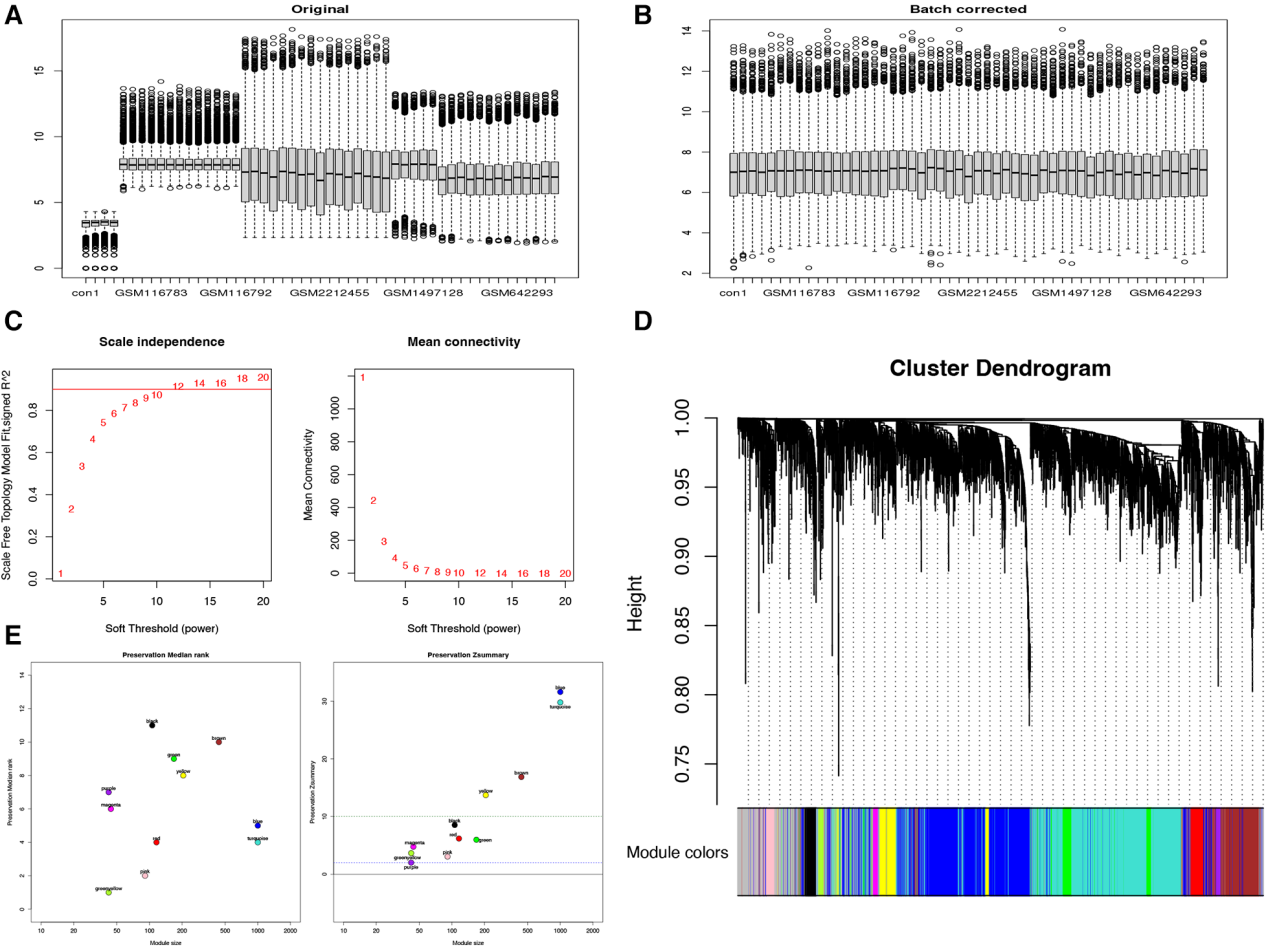
WGCNA reveals gene coexpression network in 28 patients with TAA. (**A**) Before and (**B**) after de-batching. (**C**) Analysis of the scale-free fit index (left) and mean connectivity (right) for different soft-thresholding powers. (**D**) Clustering dendrogram of TAA-related DEGs. (**E**) Module reproducibility.

To investigate potential differential expression patterns during TAA formation and development, we utilized the WGCNA method, employing an unordered network to modularize and enrich the genes of 28 patients with TAA (Figure 1). The genes were classified based on their respective expression levels; after batch processing (Figures 1A,B), we determined that a soft threshold (*β*) value of 12 yielded the most suitable connectivity between genes in the gene network (Figure 1C). Subsequently, 11 coexpressed modules were identified, with the gray module containing genes that were not assigned to any module (Figure 1D). Among these modules, the blue, turquoise, brown, and yellow modules exhibited Zsummary statistics >10 and exhibited the highest stability. The blue and turquoise modules also demonstrated relatively small median rank statistics, indicating relatively good repeatability (Figure 1E) and a positive correlation with TAA.

### 3.2 Correlation analysis between module characteristics and TAA

**Figure 2:**
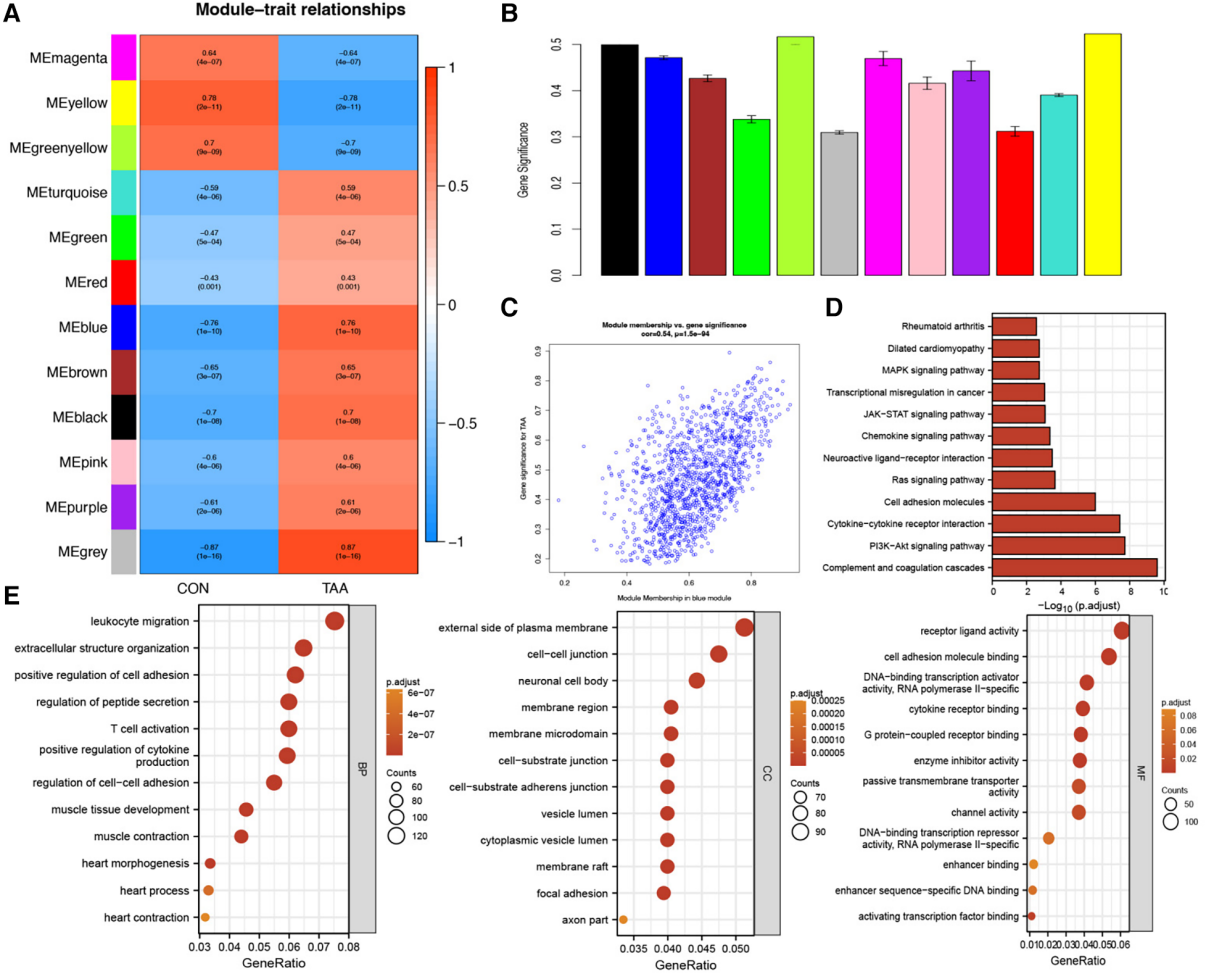
Module–trait correlation analyses. (**A**) Heatmap showing the correlation between modules and TAA. (**B**) Module significance values of coexpression modules associated with TAA. (**C**) Gene significance for TAA in the blue module. (**D**,**E**) KEGG pathway and GO functional enrichment analysis of hub genes.

To further validate the relevance of each gene module to TAA, we performed a correlation analysis between each module and TAA. Based on the heatmap of the correlation between the modules and TAA and the module significance map of the coexpression modules related to TAA, we chose the MEblue module for subsequent analyses. We analyzed the correlation between the genes in the blue module and TAA (Figure 2C). Utilizing three criteria, i.e., correlation with TAA >0.3, gene significance >0.3, and module membership >0.7, we identified hub genes, which were then subjected to KEGG pathway and GO functional enrichment analysis. The hub genes were found to be significantly enriched in pathways such as complement and coagulation cascades, PI3K–Akt signaling pathway, cytokine–cytokine receptor interaction, cell adhesion molecules, Ras signaling pathway, neuroactive ligand–receptor interaction, and chemokine signaling pathway (Figure 2D). Furthermore, the hub genes were significantly enriched in diverse biological processes, such as leukocyte migration, extracellular structure organization, positive regulation of cell adhesion, regulation of peptide secretion, T cell activation, positive regulation of cytokine production, muscle tissue development, and muscle contraction. The cellular components enriched included, for example, extracellular side of plasma membrane, neuronal cell body, membrane region, membrane microdomain, cell–substrate junction, cell–substrate adherens junction, vesicle lumen, and cytoplasmic vesicle lumen, and the molecular functions enriched included, for example, receptor ligand activity; cell adhesion molecule binding; DNA-binding transcription activator activity, RNA polymerase II-specific; cytokine receptor binding; G protein-coupled receptor binding; and enzyme inhibitor activity (Figure 2E).

### 3.3 ATAC-seq

**Figure 3:**
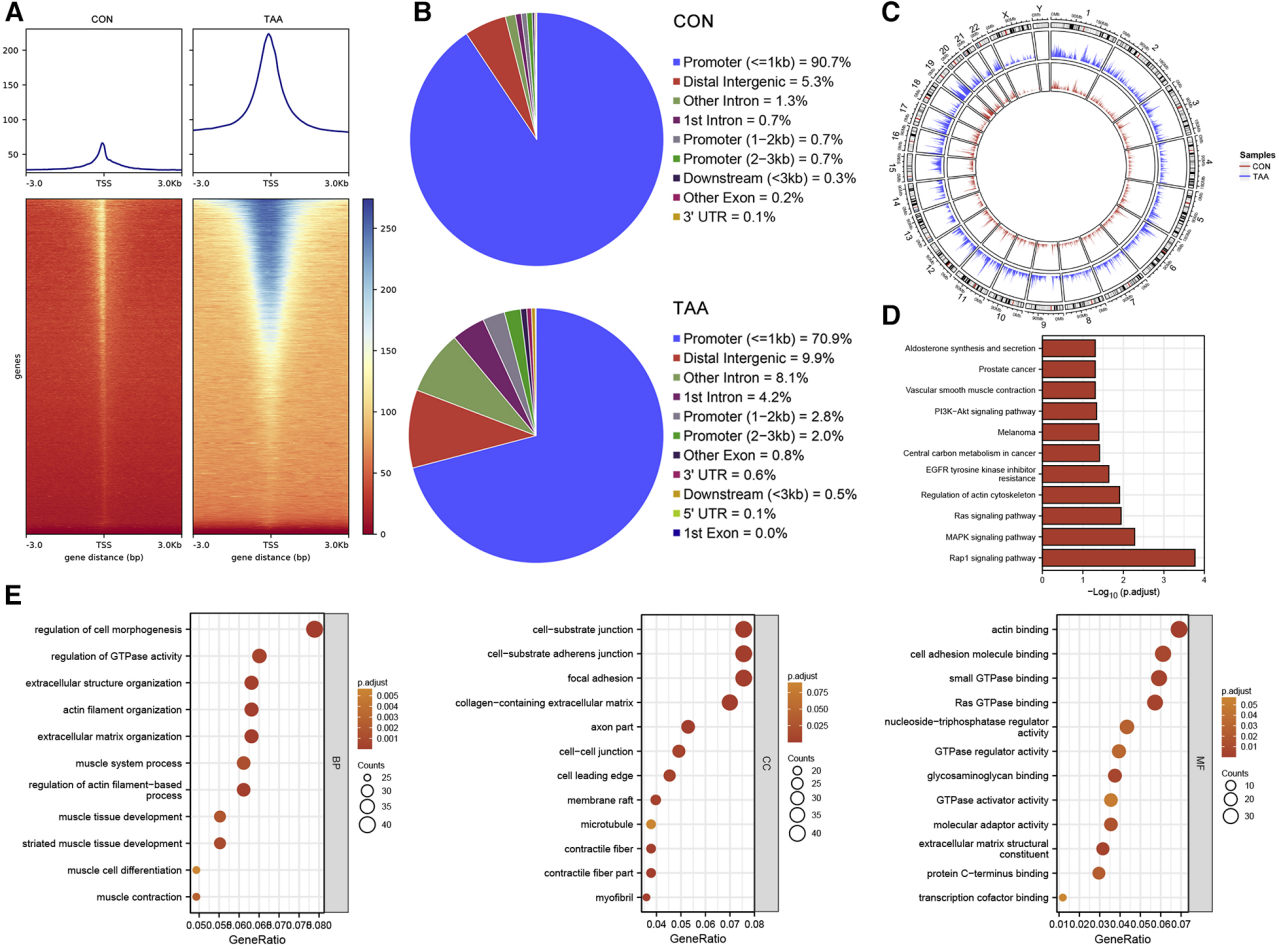
ATAC-seq results. (**A**) TSS enrichment heatmap. (**B**) Distribution proportion of chromatin accessible regions. (**C**) Circos plot, with the outer circles indicating chromosomal positions and the inner circles representing different samples. (**D**,**E**) KEGG and GO functional enrichment analysis of transcription factor binding site-associated genes.

For chromatin accessibility analysis, ATAC-seq was performed on tissue samples obtained from two patients with BAV and control tissue samples obtained from two heart transplant donors. The determined accessible regions were observed to be mainly enriched within 3 kb of the TSS (Figure 3A), and in the presumed accessible regions, >70% promoters were located 1 kb upstream of the TSS (Figure 3B). Moreover, the distribution and enrichment of all peaks on chromosomes, which were plotted using Circos, indicated that BAV-associated TAA chromatin accessibility was generally higher than that of the control group, possibly reflecting increased cell type diversity in TAA (Figures 3A–C). KEGG pathway and GO functional enrichment analyses of transcription factor binding site-associated genes revealed significant enrichment in pathways such as Rap1 signaling pathway, MAPK signaling pathway, Ras signaling pathway, regulation of actin cytoskeleton, and EGFR tyrosine kinase inhibitor resistance (Figure 3D); furthermore, the associated genes were significantly enriched in various biological processes, such as regulation of cell morphogenesis, regulation of GTPase activity, extracellular structure organization, actin filament organization, and extracellular matrix organization. The cellular components enriched included, for example, cell–substrate junction, cell–substrate adherens junction, focal adhesion, collagen-containing extracellular matrix, and axon part, and the molecular functions enriched included, for example, actin binding, cell adhesion molecule binding, small GTPase binding, Ras GTPase binding, nucleotide-triphosphatase regulator activity, and GTPase regulator activity (Figure 3E).

### 3.4 Single-cell data processing and cell subpopulation identification

**Figure 4:**
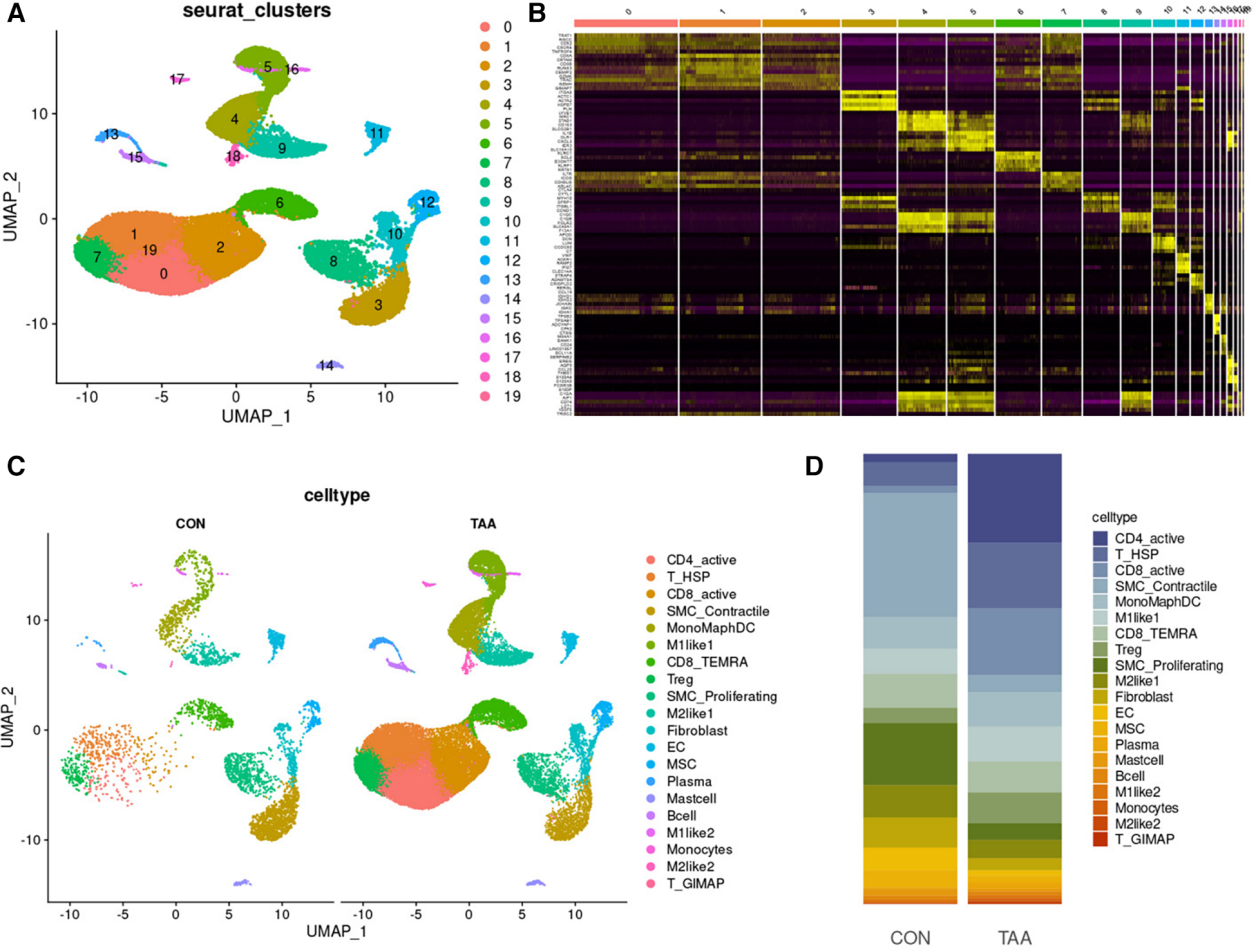
Cell-type classification in TAA. (**A**) UMAP plot of 20 cell clusters. (**B**) Heatmap of the top five marker genes in each cell cluster. (**C**) Cell distribution in TAA and control tissues. (**D**) Bar plot of the distribution of all identified cell clusters: proportion of each cell in TAA and control tissues.

Unsupervised clustering based on Seurat identified 19 different cell populations (Figure 4A). Cell subpopulations were further identified based on the top five marker genes for each cell subpopulation (Figure 4B). Comparing TAA and control samples, 20 cell subpopulations were classified according to characteristic markers (Figure 4C), and their distribution differences were analyzed (Figure 4D). Relative to the control group, the TAA group exhibited fewer SMC_Proliferating cells, SMC_Contractile cells, ECs, MSCs, M2like1s, fibroblasts, Plasma cells, Mast cells, and B cells but higher CD4_active cells, T_HSP cells, CD8_active cells, Tregs, M1like1s, M2like2s, T_GIMAPs. The relative numbers of MonoMaphDCs, CD8_TEMRAs, M1like2s, and Monocytes were unaffected. Collectively, these findings suggested that cells with considerable differences, such as CD4_active, SMC_Contractile, and SMC_Proliferating cells, are involved in the process of BAV-induced TAA onset.

### 3.5 Differential gene analysis

**Figure 5:**
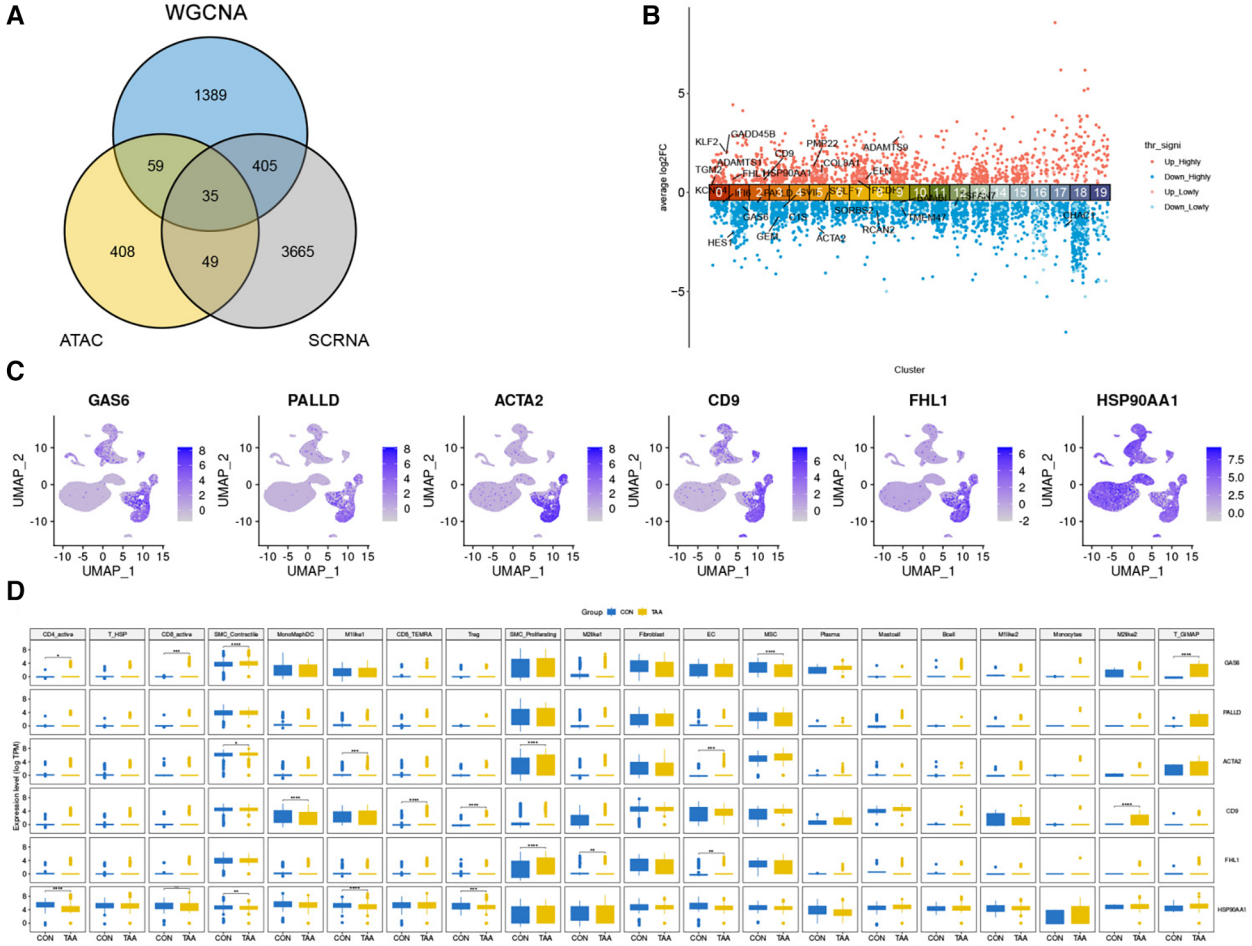
Differential genes in cell populations of TAA. (**A**) Venn diagram showing the number of differential genes identified from WGCNA and ATAC-seq, resulting in 34 genes. (**B**) DEG annotation for each cell subpopulation of TAA (adjusted *p* < 0.01 = highly significant). (**C**) UMAP plot of the six genes with higher expression levels. (**D**) Expression levels of these six genes in each cell type.

By comparing the intersection of DEGs identified from WGCNA and ATAC-seq, we obtained 34 DEGs (Figure 5A), which were subsequently annotated for each cell subpopulation of TAA (Figure 5B). Among them, the expression of CD9, FHL1y, and heat shock protein 90 alpha family class A member 1 (HSP90AA1) was upregulated and that of GAS6, PALLD, and ACTA2 was downregulated. Further investigation of their expression levels in various cell types revealed significant expression in cells such as SMC_Contractile cells, SMC_Proliferating cells, Fibroblasts, and MSCs (Figures 5C,D).

### 3.6 Pseudotemporal analysis of cell trajectory changes in TAA

**Figure 6:**
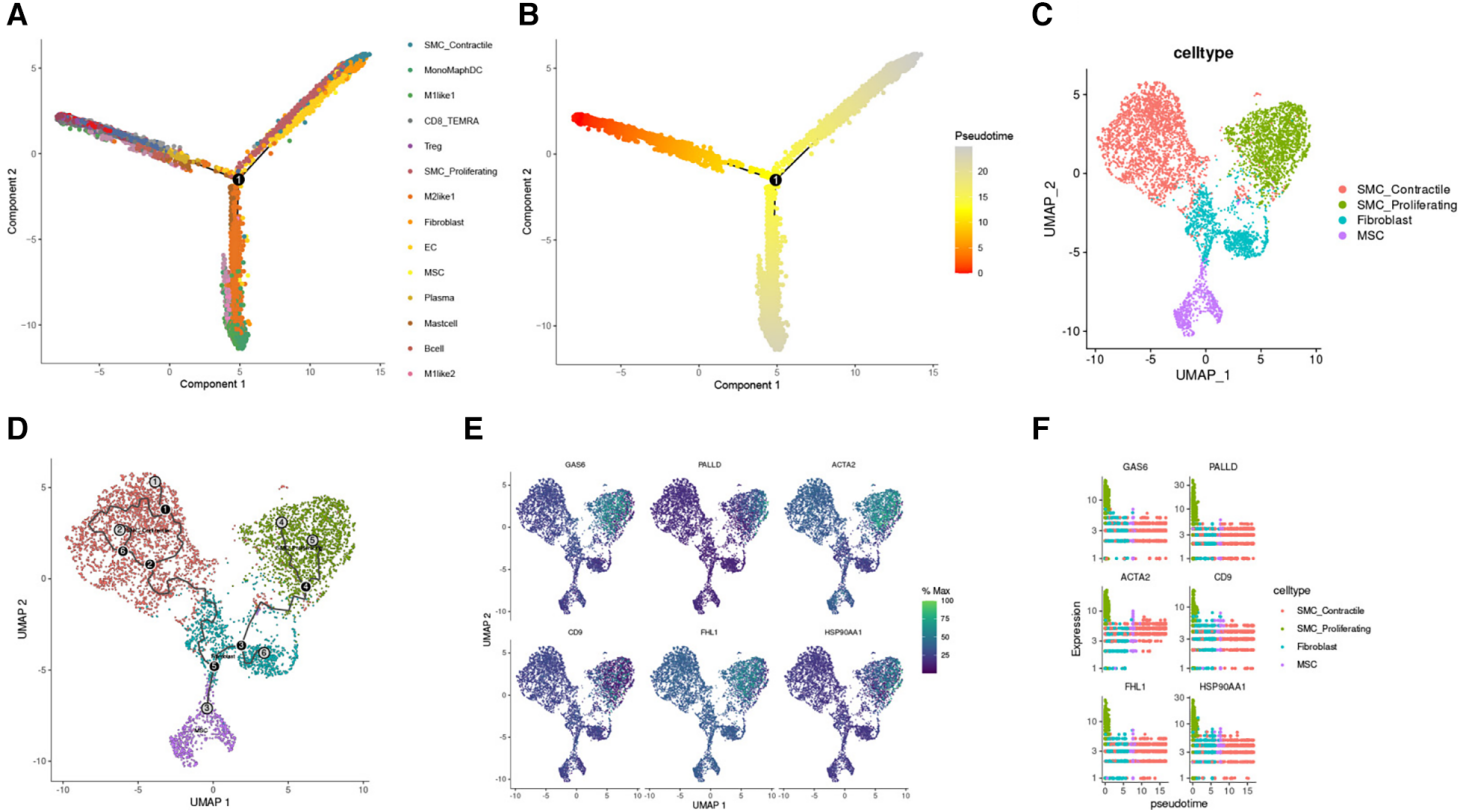
Reverse chronological analysis of single-cell samples. (**A**) Different colors represent different cell clusters. (**B**) The proposed temporal order, indicated by color from dark to light. (**C**) Partitioning of SMC Contractile, SMC Proliferating, Fibroblast, and MSC clusters using Monocle3. (**D**) Identification of cell trajectories. (**E**) FeaturePlot of the six genes mentioned above. (**F**) Gene expression trend plots for the six genes (gene expression changes along the proposed time course from start to termination).

During TAA development, cellular phenotype and functions undergo continuous changes. Pseudo-time analysis results revealed that SMC_Contractile cells were the predominant cell type in the early stage of TAA development. As the cells progressed through the branching node, SMC_Proliferating cells, Fibroblasts, and MSCs became the main cells in TAA development (Figures 6A,B). To investigate the developmental process of these cells in TAA, we performed trajectory analysis using Monocle3, sorting the clusters along the differentiation stage to visualize the process of cell differentiation in TAA (Figures 6C,D). In addition, using Monocle3, we plotted the relative expression levels of six genes to highlight their changing trends in the cell development trajectory (Figures 6E,F).

## 4 Discussion

TAA is a life-threatening condition characterized by continuous expansion and chronic progression. Approximately 20% TAAs are attributed to specific genetic mutations (7–10, 15–19). The pathogenesis of most TAAs caused by genetic defects can be linked to abnormal development or functional defects of connective tissue, as observed in Marfan syndrome and Loeys–Dietz syndrome (10, 20). In addition, thoracic aortic dilation is frequently observed in patients with BAV (4, 21).

In this study, we utilized single-cell sequencing technology to comprehensively analyze key genes and biological processes involved in the pathogenesis of BAV-associated TAA. Our analyses led to the identification of pivotal coexpression modules related to functional pathways as well as cells that play a crucial role in TAA development, including SMC_Contractile cells, SMC_Proliferating cells, Fibroblasts, and MSCs. Furthermore, genes that were markedly up- (CD9, FHL1y, and HSP90AA1) or downregulated (GAS6, PALLD, and ACTA2) were identified. Our comprehensive bioinformatics analysis also led to the identification of differentially expressed transcription factors, downstream marker pathways/immune-related pathways, and immune cells, highlighting key immune-related genes in BAV-associated TAA.

Herein we observed significant differences in the expression of CD9 (22) in MonoMaphDCs, CD8_TEMRAs, Tregs, and M2like2s. CD9 plays a chief role in various biological activities, including cell adhesion, migration, metastasis, growth, signal transduction, and differentiation; moreover, it participates in disease regulation and mediation. On the other hand, FHL1 (23, 24) showed significant differences in its expression in SMC_Proliferating cells, M2like1s, and ECs. This could be attributed to its role in inhibiting the proliferation of aortic vascular SMCs and influencing aortic wall remodeling. HSP90AA1 (25–27) showed significant differences in its expression in CD4_active cells, CD8_active cells, SMC_Contractile cells, M1like1s, and Tregs. HSP90AA1 is a critical molecular chaperone that is highly conserved in evolution and expressed under the stimulatory circumstances of trauma, infection, and tumors. It is extracellularly expressed and involved in several cell protection mechanisms. Besides, HSP90AA1 participates in tumor progression and cancer cell invasion, and is known to promote cancer cell proliferation and metastasis. In the pathogenesis of TAA, the expression of GAS6 (28) showed significant differences in CD4_active cells, CD8_active cells, SMC_Contractile cells, MSCs, and T_GIMAPs. GAS6 is a cytokine that is associated with various biological processes, including cell proliferation, apoptosis, migration, and inflammatory response, and expressed in many cell types, including ECs, vascular SMCs, and fibroblasts. It also plays a vital role in the occurrence and development of aortic aneurysms by activating the Axl receptor; furthermore, GAS6 affects aortic aneurysm formation by regulating platelet activation and coagulation function. GAS6 protein expression was originally found to be upregulated in growth-arrested fibroblasts. GAS6 stimulation has been reported to rescue serum-starved fibroblasts and vascular SMCs from apoptosis. PALLD (29) encodes a cytoskeletal protein involved in actin reorganization and plays an important role in heart development. ACTA2 (1, 30) expression showed a significant difference in SMC_Contractile cells, M1like1s, SMC_Proliferating cells, and ECs. ACTA2 encodes α-2 smooth muscle actin, which is involved in cell migration and muscle contraction. In aortic aneurysms, mutations in ACTA2 have been linked to weakening and expansion of the aortic wall. ACTA2 is the most common mutation gene responsible for TAA and dissection. Specific ACTA2 mutations have also been linked to an increased risk of early-onset stroke or coronary artery disease.

GO enrichment analysis revealed that associated genes were involved in diverse biological processes, including cell proliferation, indicating extensive proliferation and activation of immune cells during the pathogenesis of aortic aneurysm. Moreover, these genes were enriched in molecular functions related to extracellular matrix organization, suggestive of potential weakening of the aortic structure due to alterations in the extracellular matrix, making it more susceptible to expansion and rupture.

A limitation of this study is the small sample size, where only two patients and two controls were included. Despite this, our data reveal the cellular and molecular landscape of BAV at the single-cell level, providing valuable insights into TAA tissue cell morphology and cellular matrix structure. Furthermore, our findings offer a molecular profile of multiple isoforms of aortic SMCs, fibroblasts, macrophages, and T lymphocytes, indicating a potentially critical role of CD9, FHL1y, HSP90AA1, GAS6, PALLD, and ACTA2 genes in BAV improvement. We believe that our results enhance our understanding of the pathogenesis of BAV and may contribute to the development of new therapeutic approaches.

In this study, the data of the public dataset GSE5180, GSE83675, GSE61128 and GSE26155 were used to obtain genes significantly related to the onset of TAA patients, and then the self-test data were used to obtain significant genes related to BAV patients. By comparing the two, we identified the key immune-related genes of BAV-related TAA, and clarified the cell populations that may be involved in the pathogenesis process and their developmental trajectories. Through this study, we revealed the cellular and molecular landscape of the bicuspid aortic valve at the single-cell level, illustrated the dynamic changes of cells in terms of morphological structure and functional properties during the pathogenesis of BAV, illustrated the dynamic changes of chromatin during the pathogenesis of BAV, and suggested the potential roles of CD9, FHL1y, HSP90AA1, GAS6, PALLD and ACTA2 in BAV. These findings suggest that CD9 and other genes mediate the development regulation of BAV through cell adhesion, motility, metastasis, growth, signal transduction, differentiation and other functions. FHL1y may be involved in inhibiting the proliferation of aortic vascular SMCs and affecting aortic wall remodeling, thereby participating in the development of BAV; HSP90AA1 is stimulated to express during the pathogenesis of BAV, which may participate in the proliferation and metastasis of cells and affect BAV; GAS6 may participate in the occurrence and development of BAV by affecting fibroblasts and apoptosis of vascular smooth muscle cells. PALLD may regulate BAV through regulation of the actin cytoskeleton; ACTA2 encodes smooth muscle α-2 actin, which affects the expansion of the aorta wall and thus BAV development.

In conclusion, we performed a comprehensive analysis of the dynamics of cellular and molecular changes during BAV development. Our study identified SMCs, Fibroblast, and MSCs as the main sources of cells affecting BAV. In addition, we demonstrate that a variety of potential genes such as CD9, FHL1y, HSP90AA1, GAS6, PALLD, and ACTA2 play a role in the development of BAV, which may provide novel research ideas for slowing the progression of BAV and preventing aortic rupture. Overall, our dataset provides a valuable resource for further exploration of the pathogenesis of BAV.

The results of the present study comprised of a comprehensive bioinformatics view of BAV development. Overall, this could shed light on the mechanisms of BAV, which could lead to improved diagnosis and treatments for patients with BAV. CD9, FHL1y, HSP90AA1, GAS6, PALLD, and ACTA2 are closely related to the occurrence and development of BAV, and are expected to become new biomarkers and therapeutic targets for BAV.

## Data Availability

The datasets presented in this study can be found in online repositories. The names of the repository/repositories and accession number(s) can be found in the article/Supplementary Material.
