# Supplementary material for: Single-cell RNA sequencing and ATAC sequencing identify novel biomarkers for bicuspid aortic valve-associated thoracic aortic aneurysm
Source: Front Cardiovasc Med. 2024 Apr 8;11:1265378. doi: 10.3389/fcvm.2024.1265378 (PMC11057375; doi:10.3389/fcvm.2024.1265378)
Supplement: Supplementary file 1 [file Datasheet1.pdf]

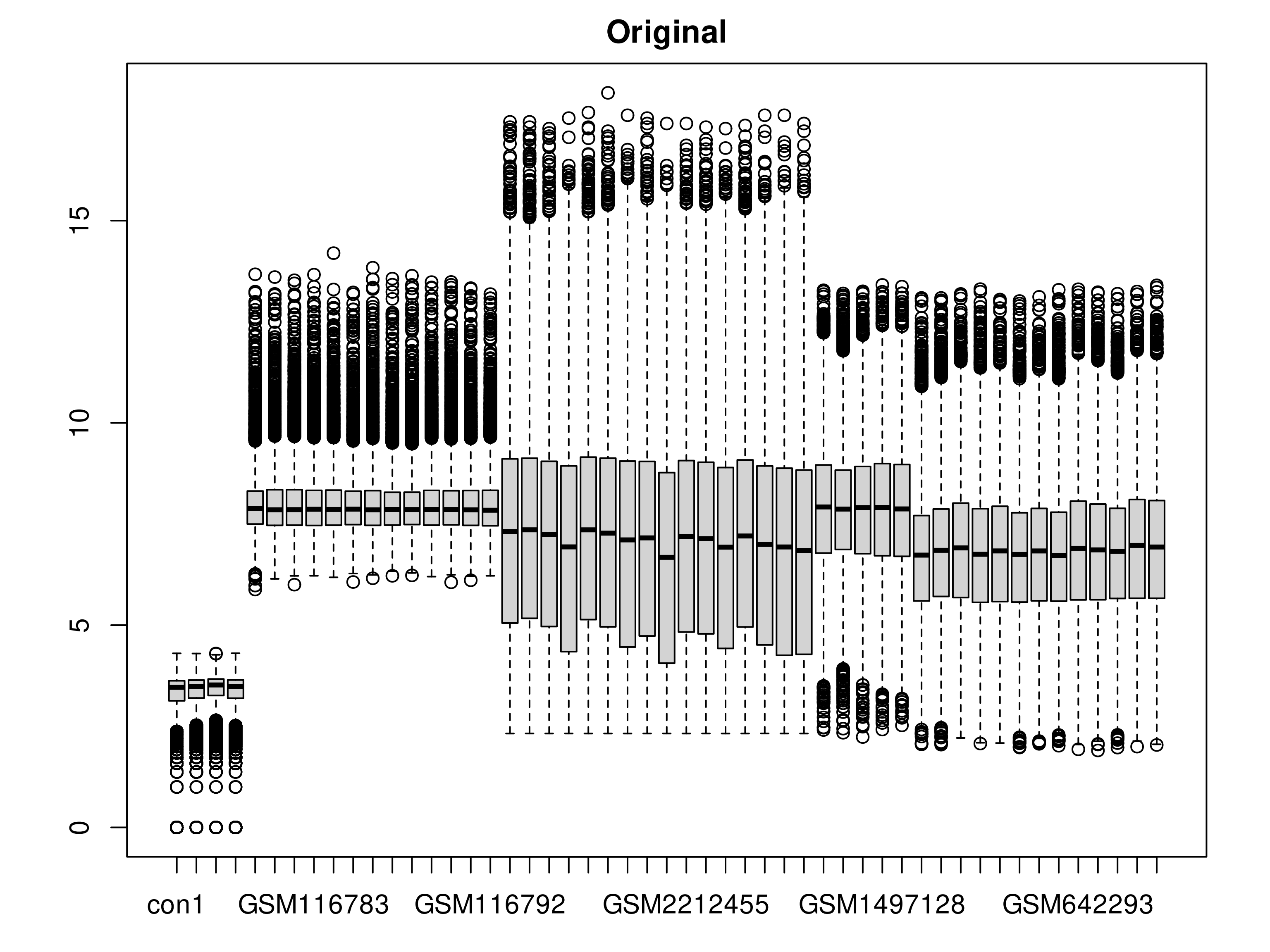
Original
15
10
5
0
con1
GSM116783
GSM116792
GSM2212455
GSM1497128
GSM642293

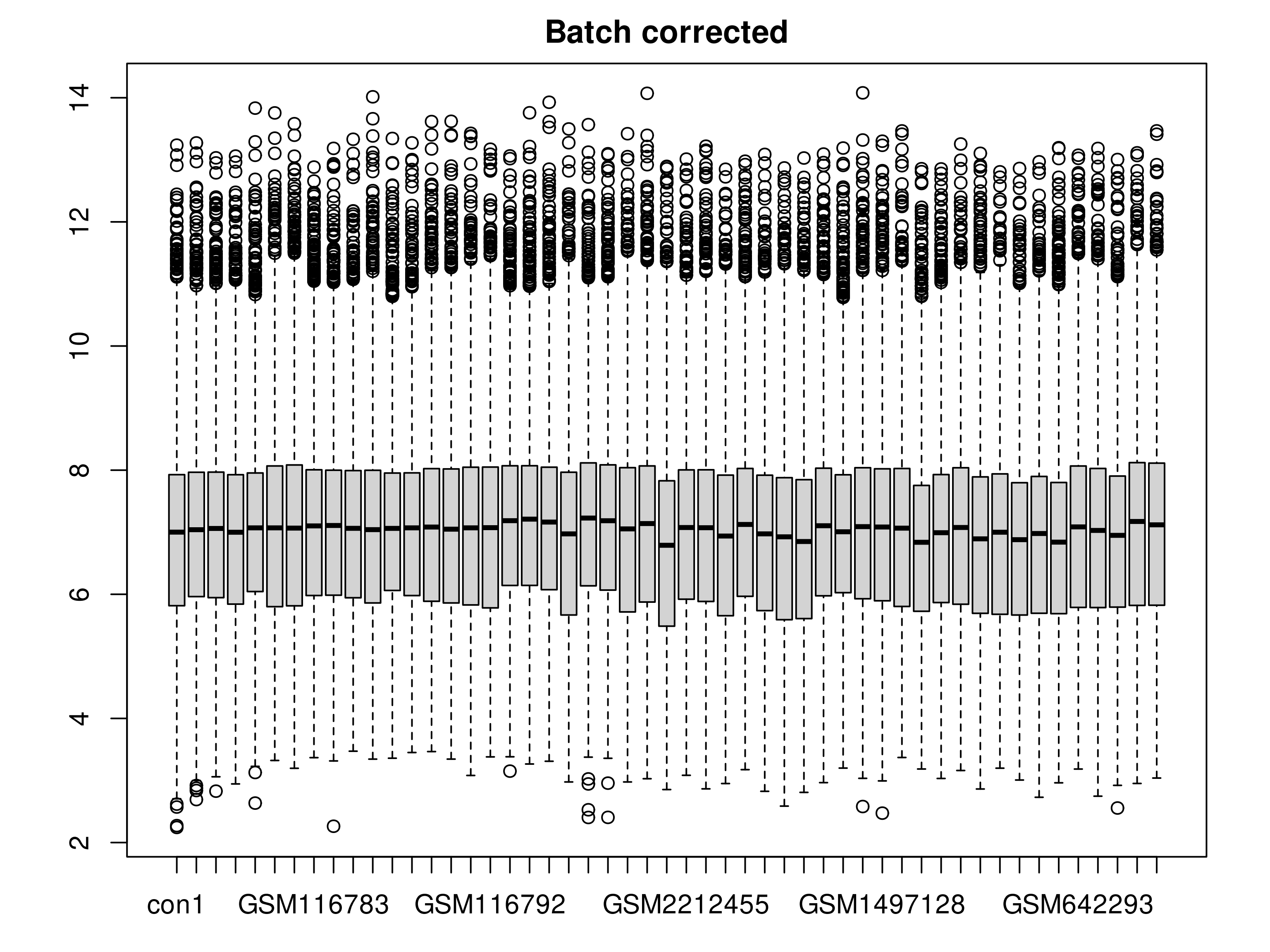
Batch corrected
14
12
10
8
6
4
2
con1
GSM116783
GSM116792
GSM2212455
GSM1497128
GSM642293

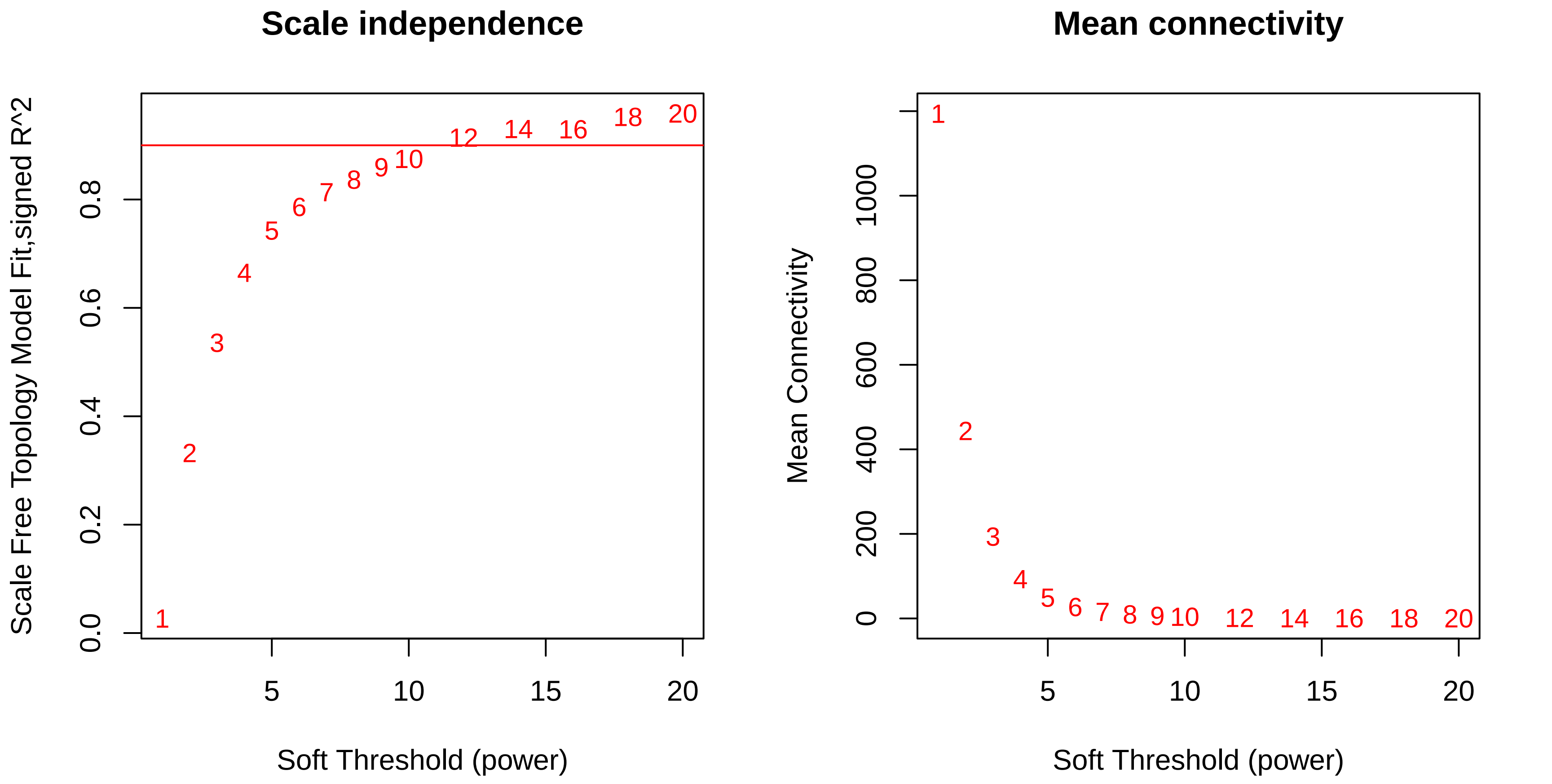

Scale independence
Scale Free Topology Model Fit,signed R^2
0.0
0.2
0.4
0.6
0.8
5
10
15
20
Soft Threshold (power)
1
2
3
4
5
6
7
8
9
10
12
14
16
18
20
Mean connectivity
Mean Connectivity
0
200
400
600
800
1000
5
10
15
20
Soft Threshold (power)

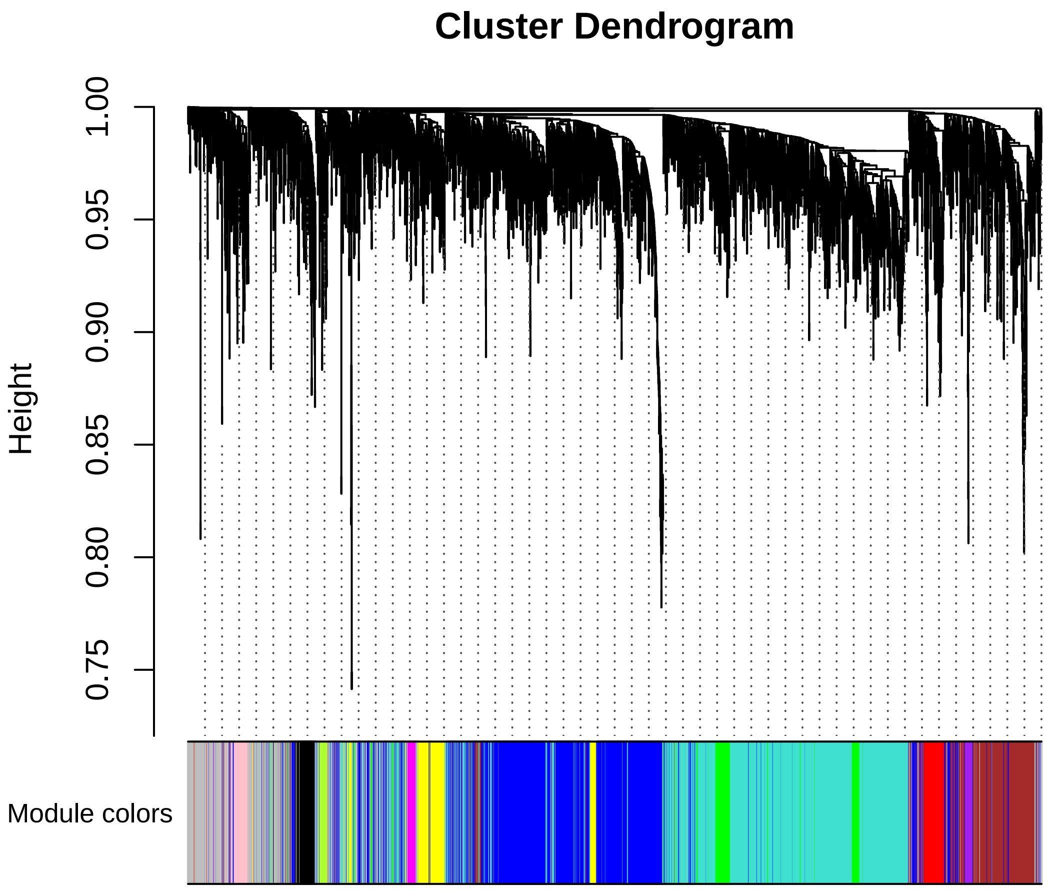

Cluster Dendrogram
Height
1.00
0.95
0.90
0.85
0.80
0.75
Module colors

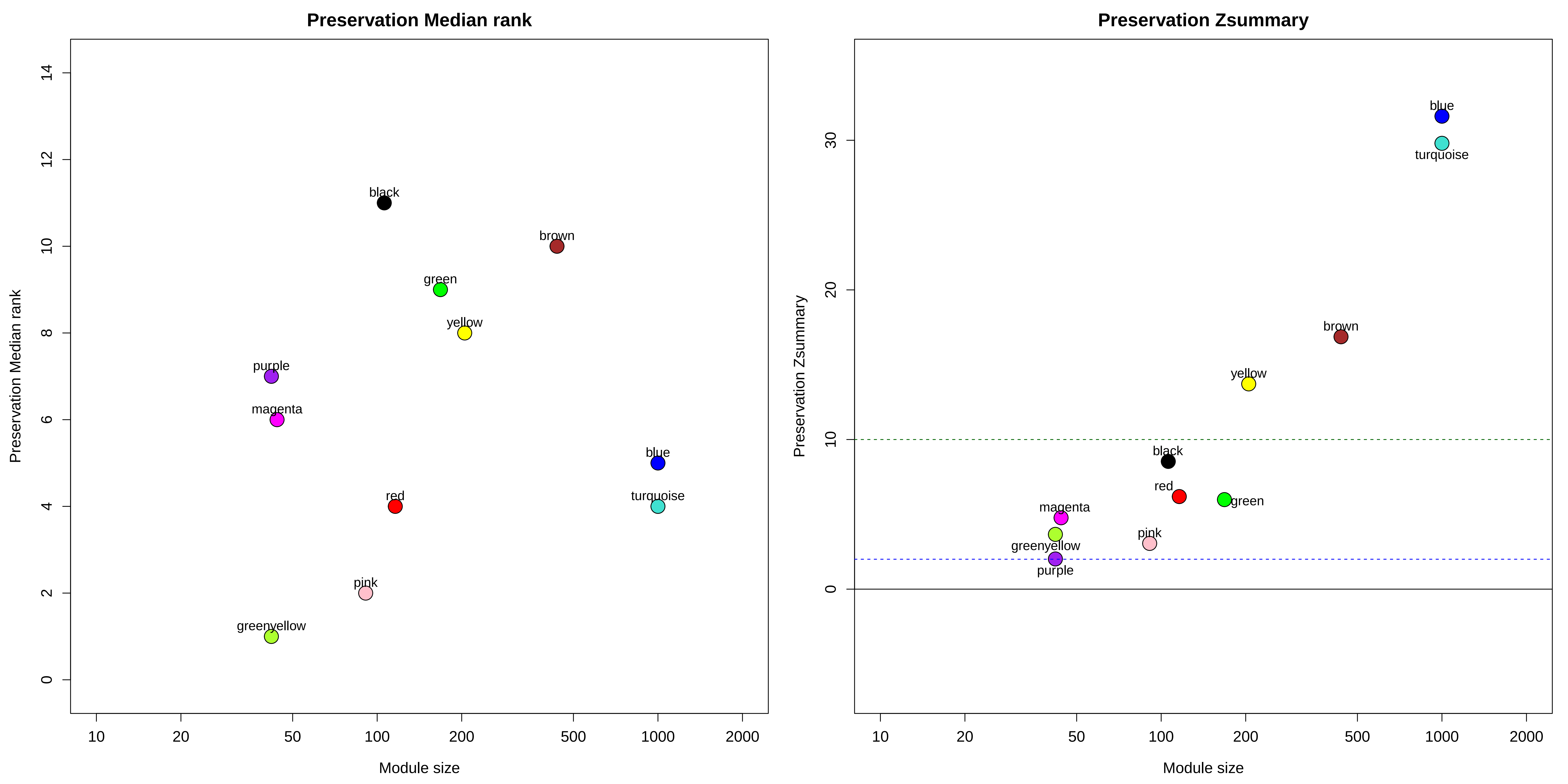
Preservation Median rank
Preservation Median rank
Module size
10
20
50
100
200
500
1000
2000
0
2
4
6
8
10
12
14
black
brown
green
yellow
purple
magenta
blue
red
turquoise
pink
greenyellow
Preservation Zsummary
Preservation Zsummary
Module size
10
20
50
100
200
500
1000
2000
0
10
20
30
blue
turquoise
brown
yellow
black
red
green
magenta
pink
greenyellow
purple
