# Supplementary material for: Single-cell RNA sequencing and ATAC sequencing identify novel biomarkers for bicuspid aortic valve-associated thoracic aortic aneurysm
Source: Front Cardiovasc Med. 2024 Apr 8;11:1265378. doi: 10.3389/fcvm.2024.1265378 (PMC11057375; doi:10.3389/fcvm.2024.1265378)
Supplement: Supplementary file 2 [file Datasheet2.pdf]

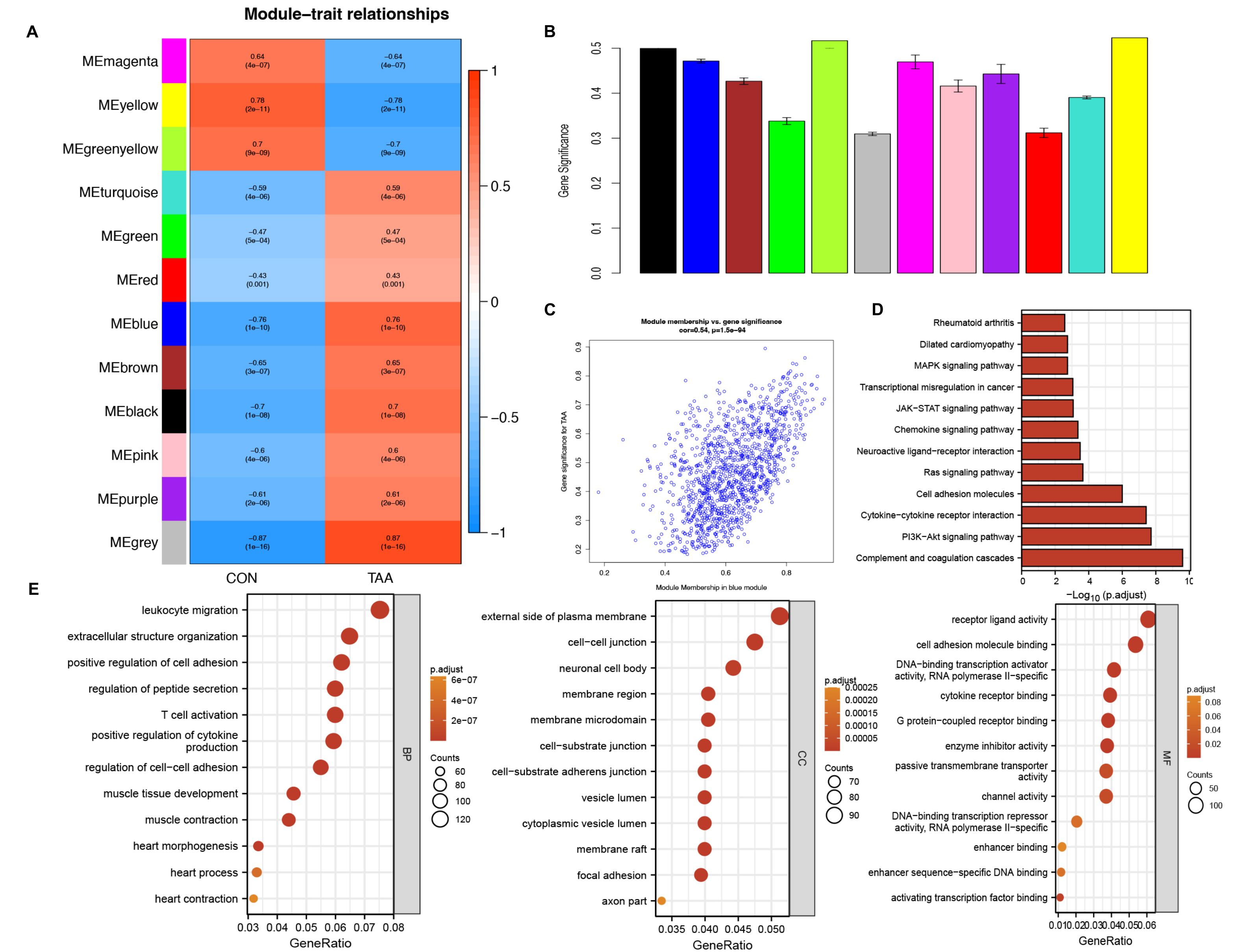

Module–trait relationships
A
MEmagenta
MEyellow
MEgreenyellow
MEturquoise
MEgreen
MEred
MEblue
MEbrown
MEblack
MEpink
MEpurple
MEgrey
0.64 (4e−07)
−0.64 (4e−07)
0.78 (2e−11)
−0.78 (2e−11)
0.7 (9e−09)
−0.7 (9e−09)
−0.59 (4e−06)
0.59 (4e−06)
−0.47 (5e−04)
0.47 (5e−04)
−0.43 (0.001)
0.43 (0.001)
−0.76 (1e−10)
0.76 (1e−10)
−0.65 (3e−07)
0.65 (3e−07)
−0.7 (1e−08)
0.7 (1e−08)
−0.6 (4e−06)
0.6 (4e−06)
−0.61 (2e−06)
0.61 (2e−06)
−0.87 (1e−16)
0.87 (1e−16)
CON
TAA
1
0.5
0
−0.5
−1
B
Gene Significance
0.0
0.1
0.2
0.3
0.4
0.5
C
Module membership vs. gene significance
cor=0.54, p=1.5e−94
Gene significance for TAA
Module Membership in blue module
D
Rheumatoid arthritis
Dilated cardiomyopathy
MAPK signaling pathway
Transcriptional misregulation in cancer
JAK−STAT signaling pathway
Chemokine signaling pathway
Neuroactive ligand−receptor interaction
Ras signaling pathway
Cell adhesion molecules
Cytokine−cytokine receptor interaction
PI3K−Akt signaling pathway
Complement and coagulation cascades
−Log10 (p.adjust)
E
leukocyte migration
extracellular structure organization
positive regulation of cell adhesion
regulation of peptide secretion
T cell activation
positive regulation of cytokine production
regulation of cell−cell adhesion
muscle tissue development
muscle contraction
heart morphogenesis
heart process
heart contraction
BP
GeneRatio
p.adjust
6e−07
4e−07
2e−07
Counts
60
80
100
120
external side of plasma membrane
cell−cell junction
neuronal cell body
membrane region
membrane microdomain
cell−substrate junction
cell−substrate adherens junction
vesicle lumen
cytoplasmic vesicle lumen
membrane raft
focal adhesion
axon part
CC
p.adjust
0.00025
0.00020
0.00015
0.00010
0.00005
Counts
70
80
90
receptor ligand activity
cell adhesion molecule binding
DNA−binding transcription activator activity, RNA polymerase II−specific
cytokine receptor binding
G protein−coupled receptor binding
enzyme inhibitor activity
passive transmembrane transporter activity
channel activity
DNA−binding transcription repressor activity, RNA polymerase II−specific
enhancer binding
enhancer sequence−specific DNA binding
activating transcription factor binding
MF
p.adjust
0.08
0.06
0.04
0.02
Counts
50
100

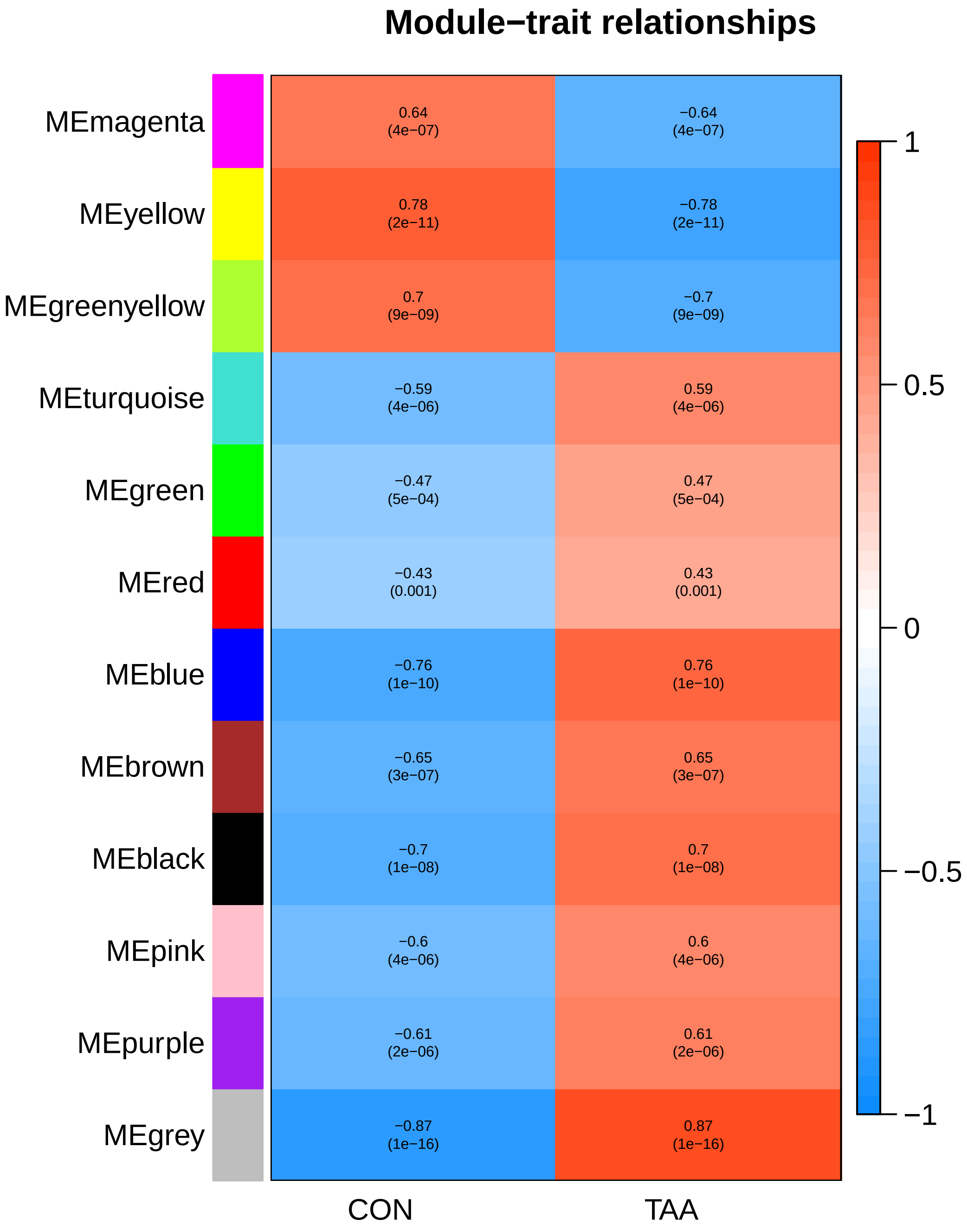
Module−trait relationships
MEmagenta
MEyellow
MEgreenyellow
MEturquoise
MEgreen
MEred
MEblue
MEbrown
MEblack
MEpink
MEpurple
MEgrey
0.64 (4e−07)
−0.64 (4e−07)
0.78 (2e−11)
−0.78 (2e−11)
0.7 (9e−09)
−0.7 (9e−09)
−0.59 (4e−06)
0.59 (4e−06)
−0.47 (5e−04)
0.47 (5e−04)
−0.43 (0.001)
0.43 (0.001)
−0.76 (1e−10)
0.76 (1e−10)
−0.65 (3e−07)
0.65 (3e−07)
−0.7 (1e−08)
0.7 (1e−08)
−0.6 (4e−06)
0.6 (4e−06)
−0.61 (2e−06)
0.61 (2e−06)
−0.87 (1e−16)
0.87 (1e−16)
CON
TAA
1
0.5
0
−0.5
−1

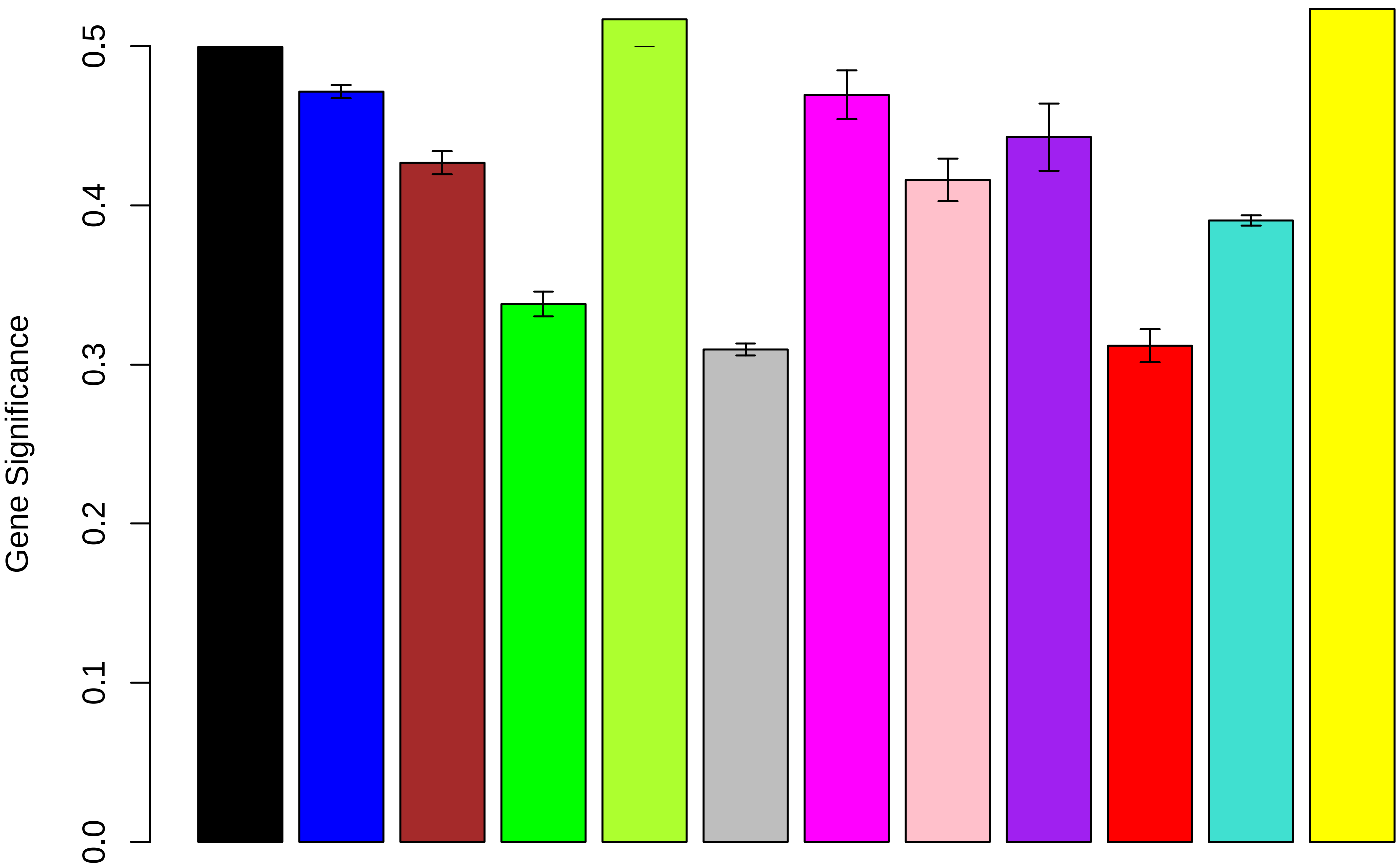
Gene Significance
0.0
0.1
0.2
0.3
0.4
0.5

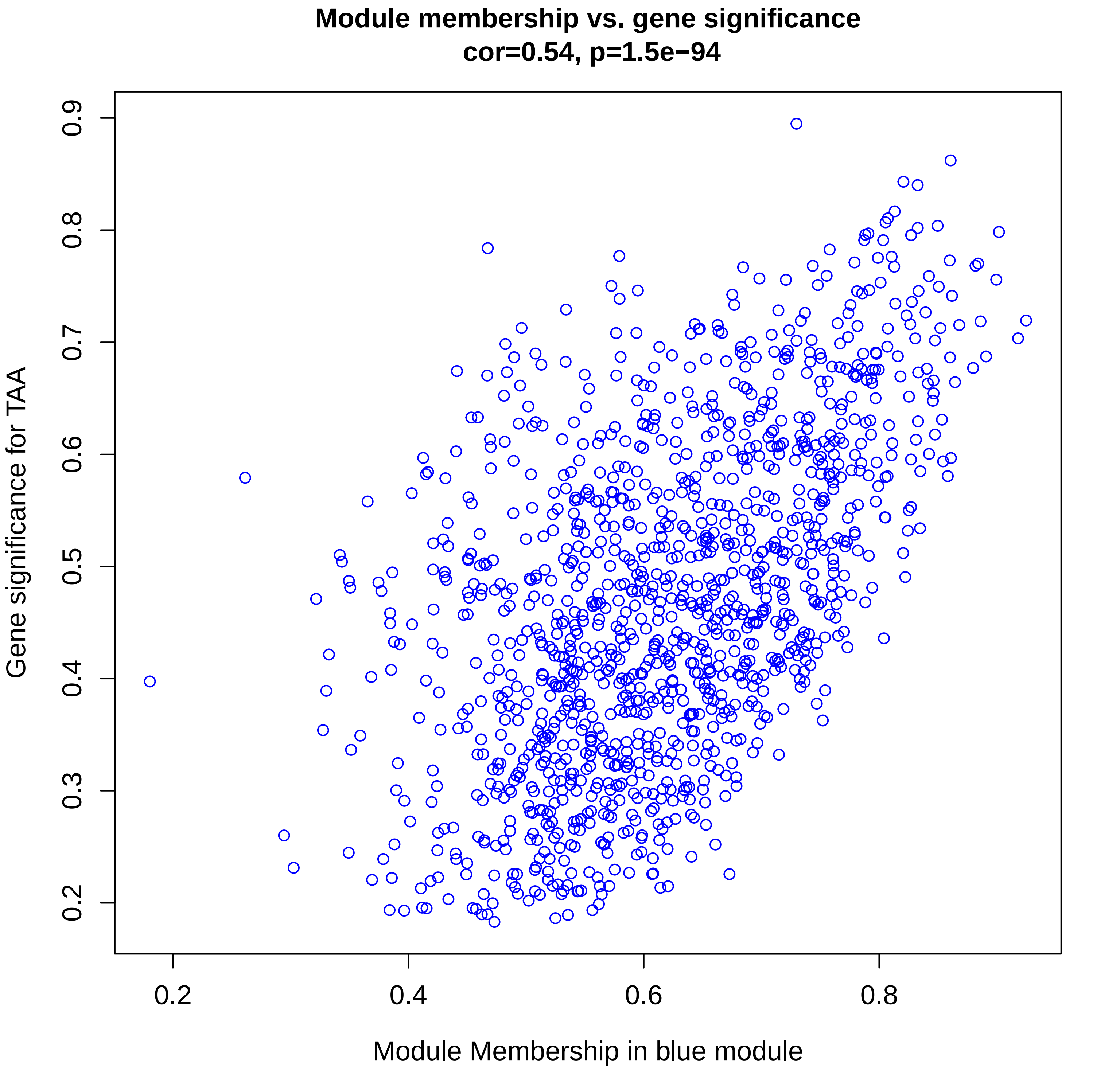
Module membership vs. gene significance
cor=0.54, p=1.5e−94
Gene significance for TAA
Module Membership in blue module
0.2
0.3
0.4
0.5
0.6
0.7
0.8
0.9
0.2
0.4
0.6
0.8

Heatmap of the module blue

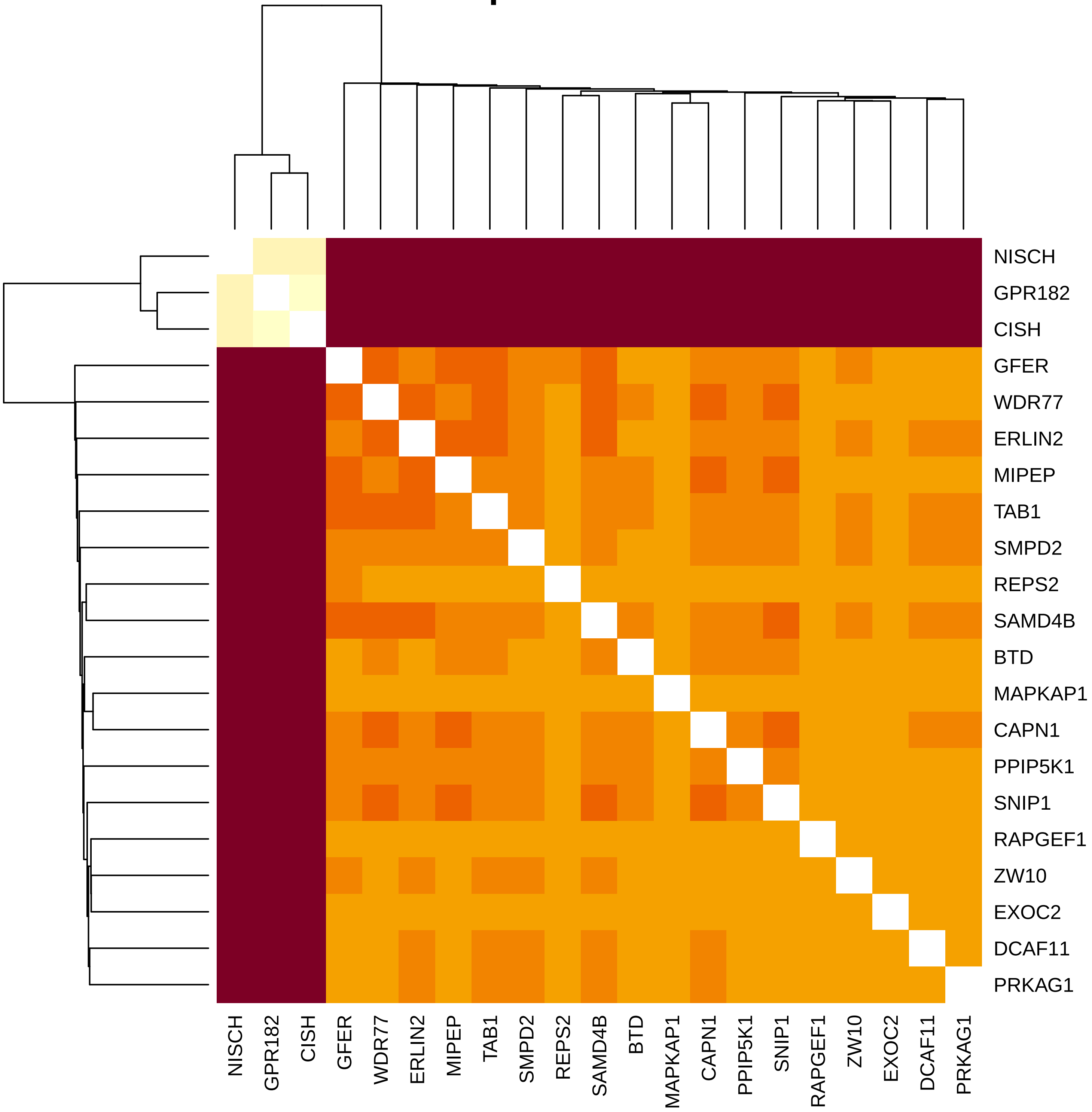

signed correlations

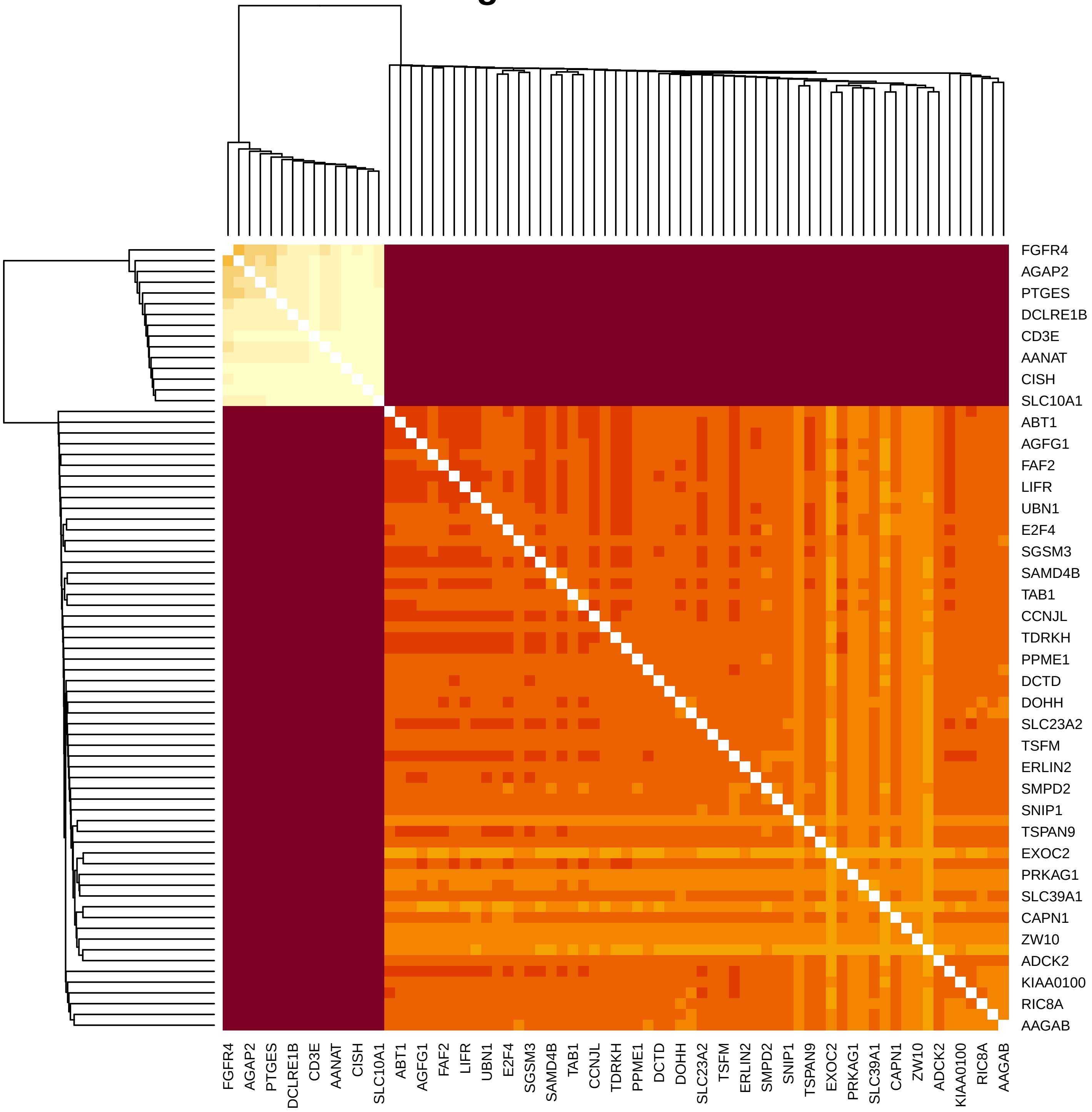

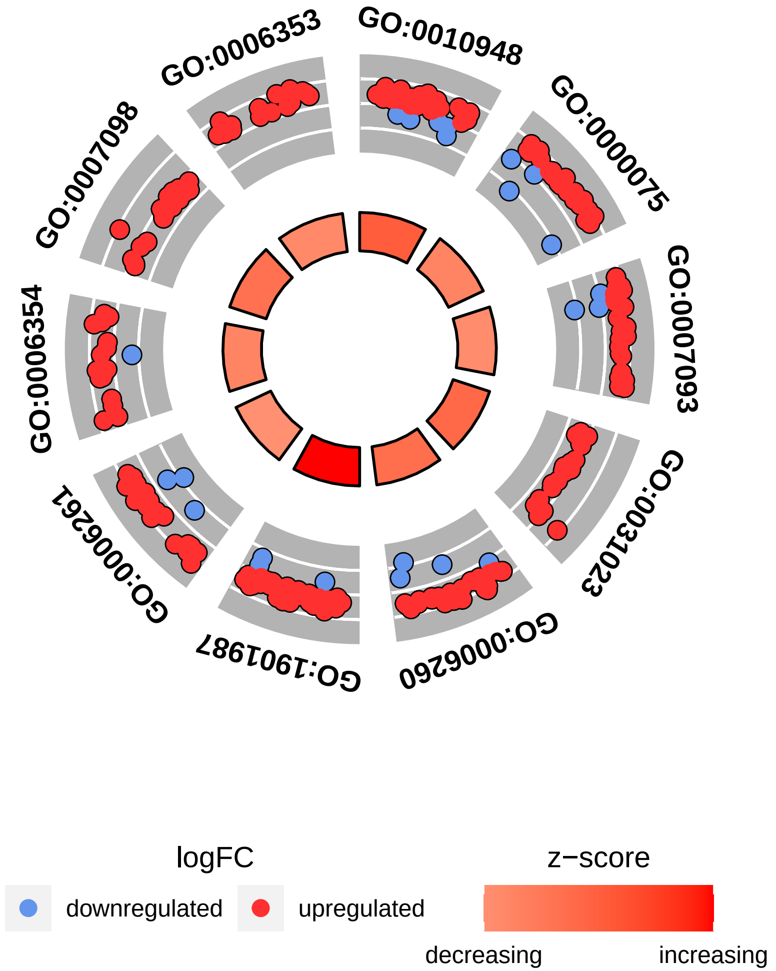

ID	Description
GO:0010948	negative regulation of cell cycle process
GO:0000075	cell cycle checkpoint signaling
GO:0007093	mitotic cell cycle checkpoint signaling
GO:0031023	microtubule organizing center organization
GO:0006260	DNA replication
GO:1901987	regulation of cell cycle phase transition
GO:0006261	DNA−dependent DNA replication
GO:0006354	DNA−templated transcription, elongation
GO:0007098	centrosome cycle
GO:0006353	DNA−templated transcription, termination

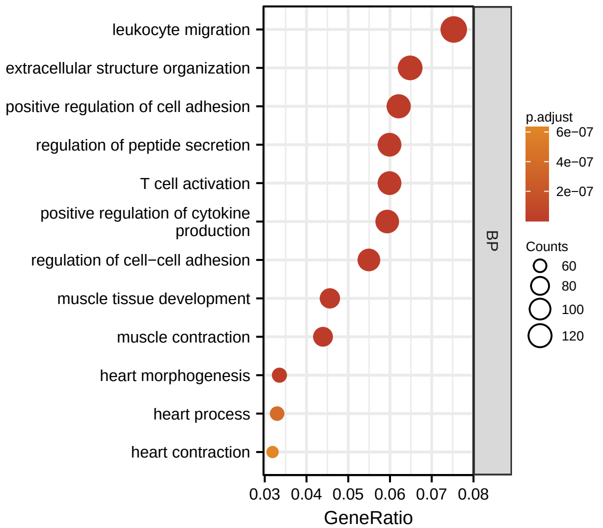
leukocyte migration
extracellular structure organization
positive regulation of cell adhesion
regulation of peptide secretion
T cell activation
positive regulation of cytokine production
regulation of cell−cell adhesion
muscle tissue development
muscle contraction
heart morphogenesis
heart process
heart contraction
0.03
0.04
0.05
0.06
0.07
0.08
GeneRatio
BP
p.adjust
6e−07
4e−07
2e−07
Counts
60
80
100
120

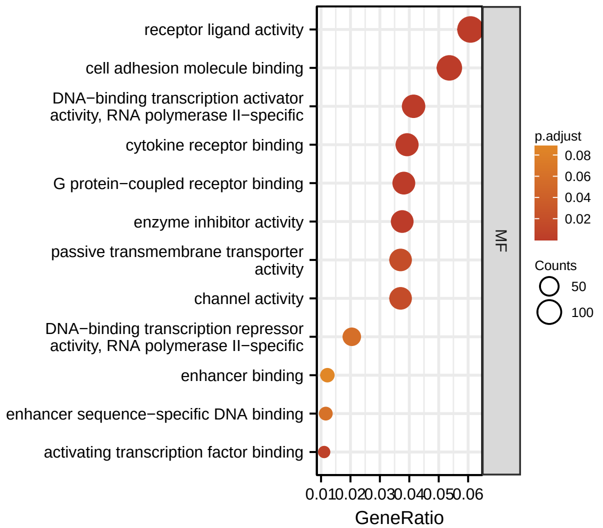
receptor ligand activity
cell adhesion molecule binding
DNA−binding transcription activator activity, RNA polymerase II−specific
cytokine receptor binding
G protein−coupled receptor binding
enzyme inhibitor activity
passive transmembrane transporter activity
channel activity
DNA−binding transcription repressor activity, RNA polymerase II−specific
enhancer binding
enhancer sequence−specific DNA binding
activating transcription factor binding
0.01
0.02
0.03
0.04
0.05
0.06
GeneRatio
MF
p.adjust
0.08
0.06
0.04
0.02
Counts
50
100

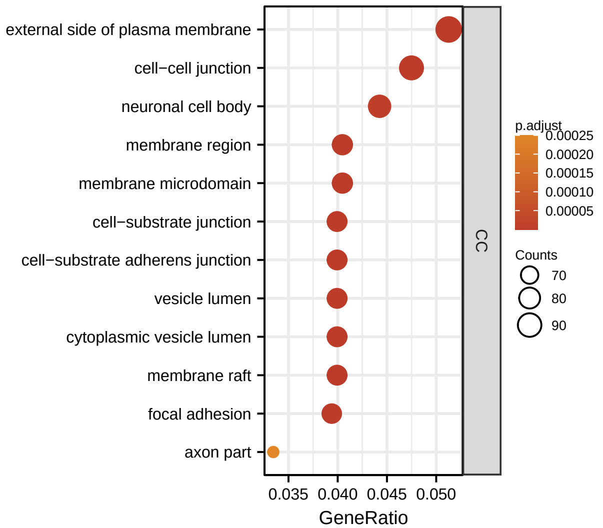
external side of plasma membrane
cell−cell junction
neuronal cell body
membrane region
membrane microdomain
cell−substrate junction
cell−substrate adherens junction
vesicle lumen
cytoplasmic vesicle lumen
membrane raft
focal adhesion
axon part
0.035
0.040
0.045
0.050
GeneRatio
CC
p.adjust
0.00025
0.00020
0.00015
0.00010
0.00005
Counts
70
80
90

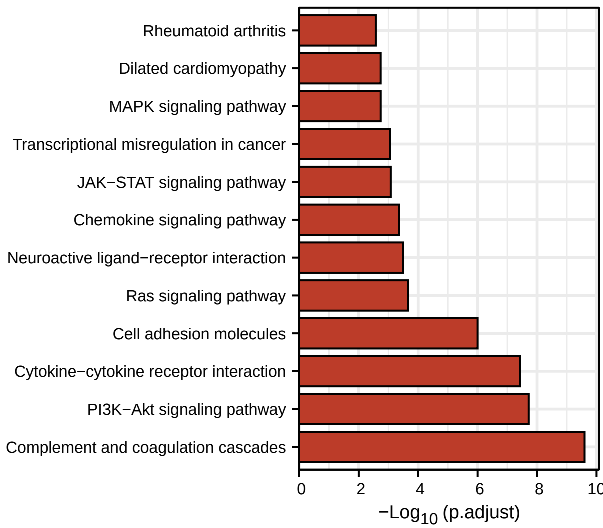
Rheumatoid arthritis
Dilated cardiomyopathy
MAPK signaling pathway
Transcriptional misregulation in cancer
JAK−STAT signaling pathway
Chemokine signaling pathway
Neuroactive ligand−receptor interaction
Ras signaling pathway
Cell adhesion molecules
Cytokine−cytokine receptor interaction
PI3K−Akt signaling pathway
Complement and coagulation cascades
0
2
4
6
8
10
−Log10 (p.adjust)

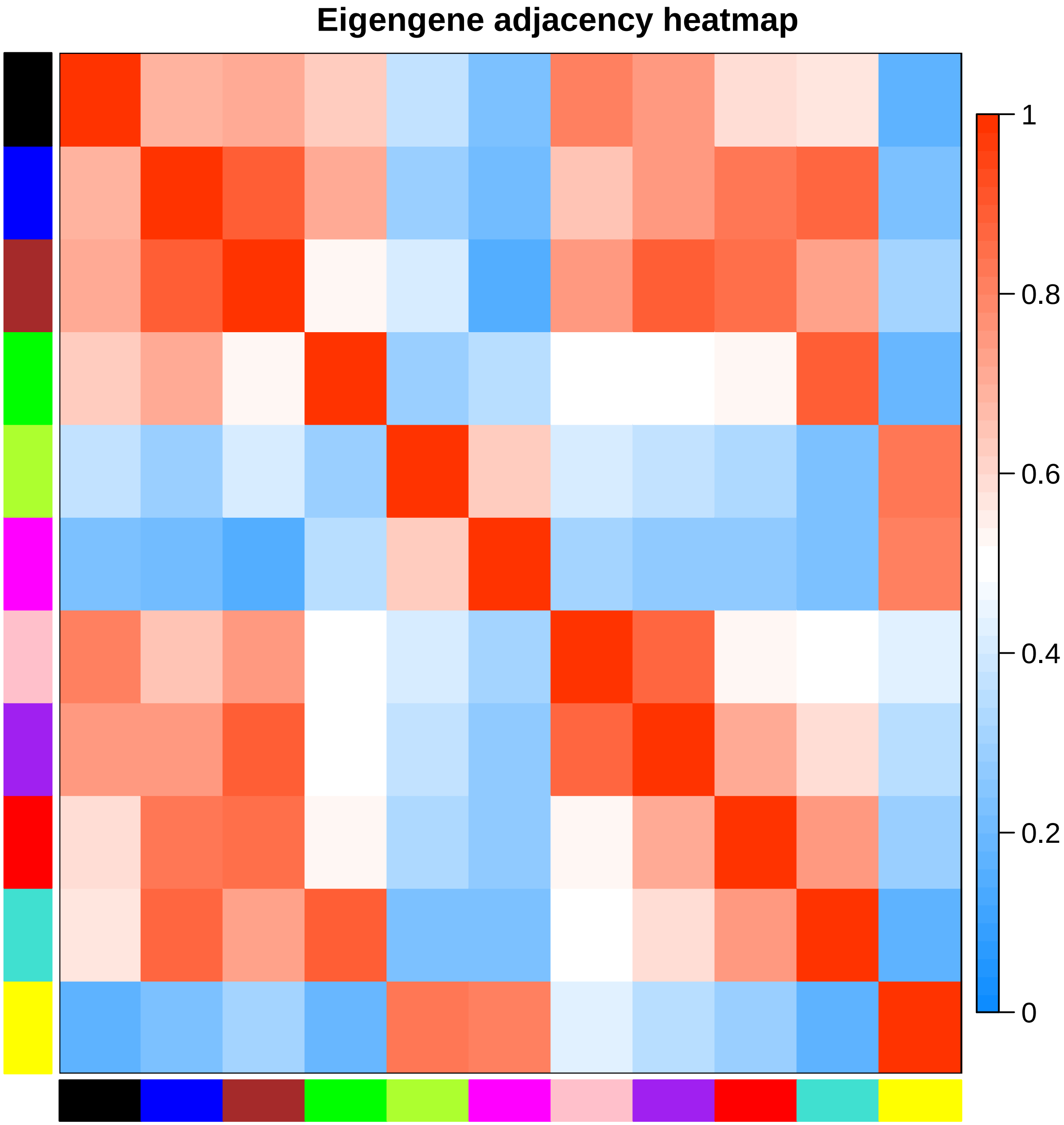
Eigengene adjacency heatmap
1
0.8
0.6
0.4
0.2
0

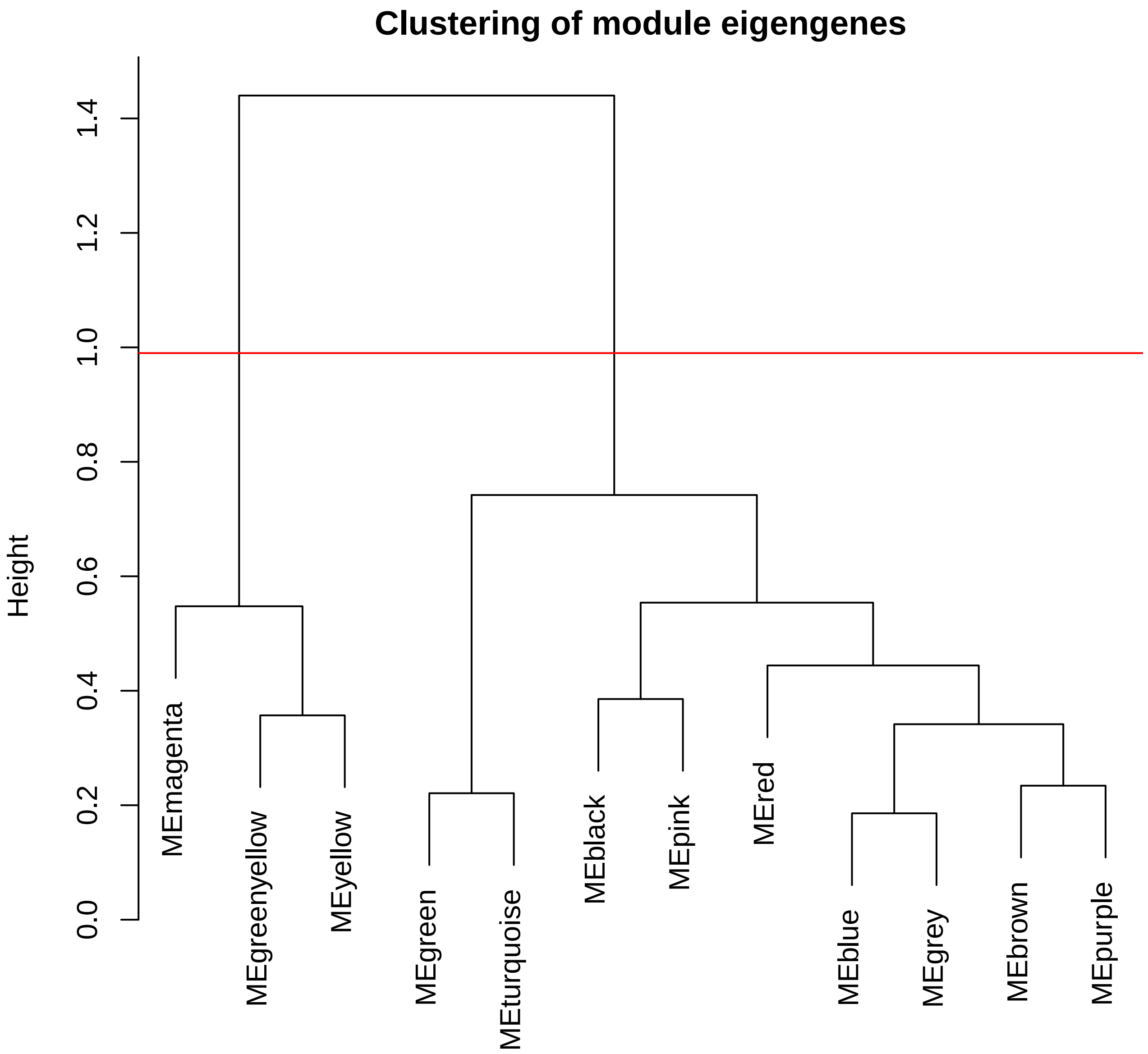
Clustering of module eigengenes
Height
0.0
0.2
0.4
0.6
0.8
1.0
1.2
1.4
MEmagenta
MEgreenyellow
MEyellow
MEgreen
MEturquoise
MEblack
MEpink
MEred
MEblue
MEgrey
MEbrown
MEpurple
