# Supplementary figures and images for: Single-cell RNA sequencing and ATAC sequencing identify novel biomarkers for bicuspid aortic valve-associated thoracic aortic aneurysm
Source: Front Cardiovasc Med. 2024 Apr 8;11:1265378. doi: 10.3389/fcvm.2024.1265378 (PMC11057375; doi:10.3389/fcvm.2024.1265378)

CON

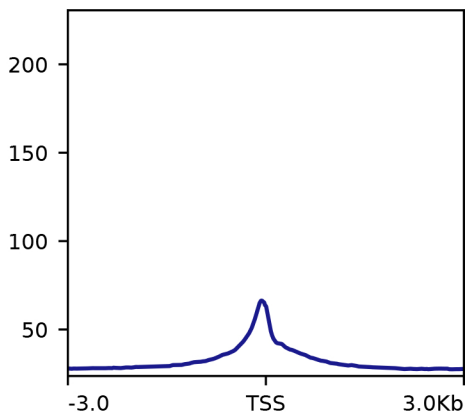

TAA

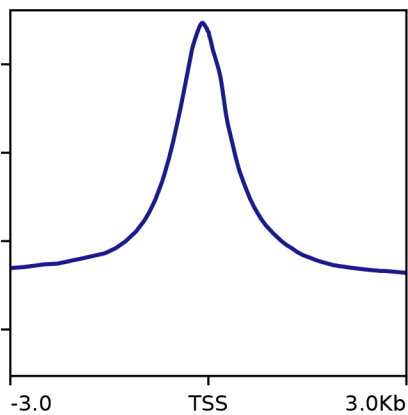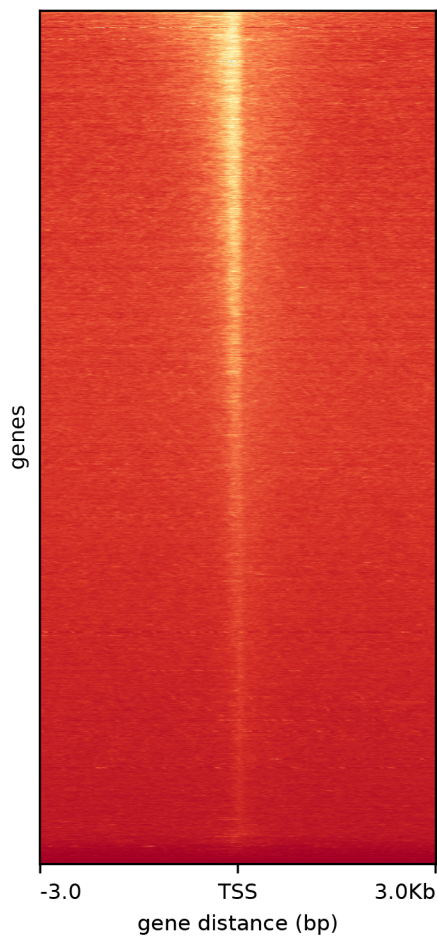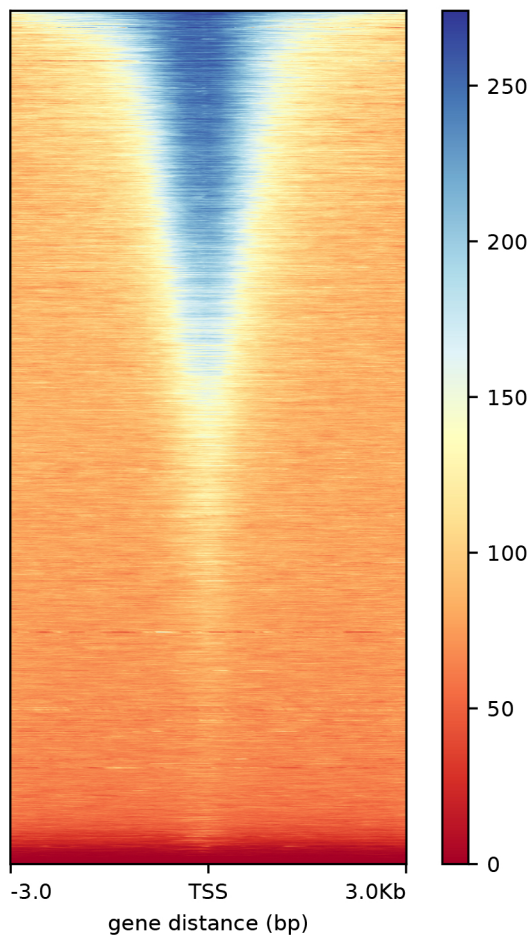

## Peaks Distribution

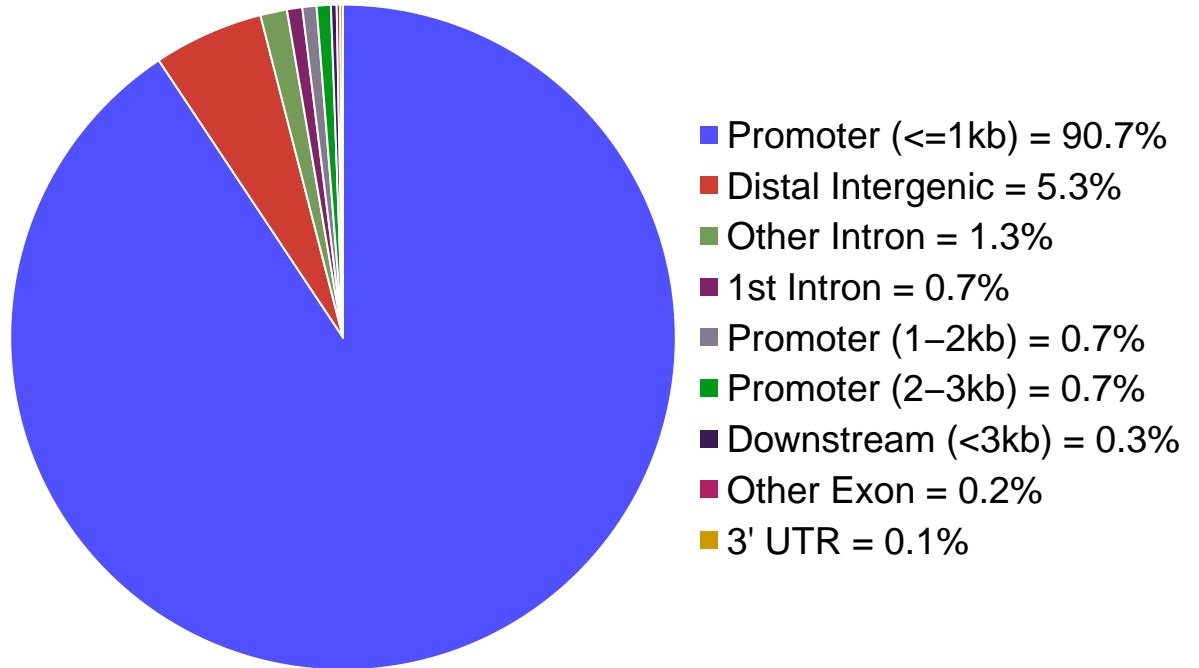

## Peaks Distribution

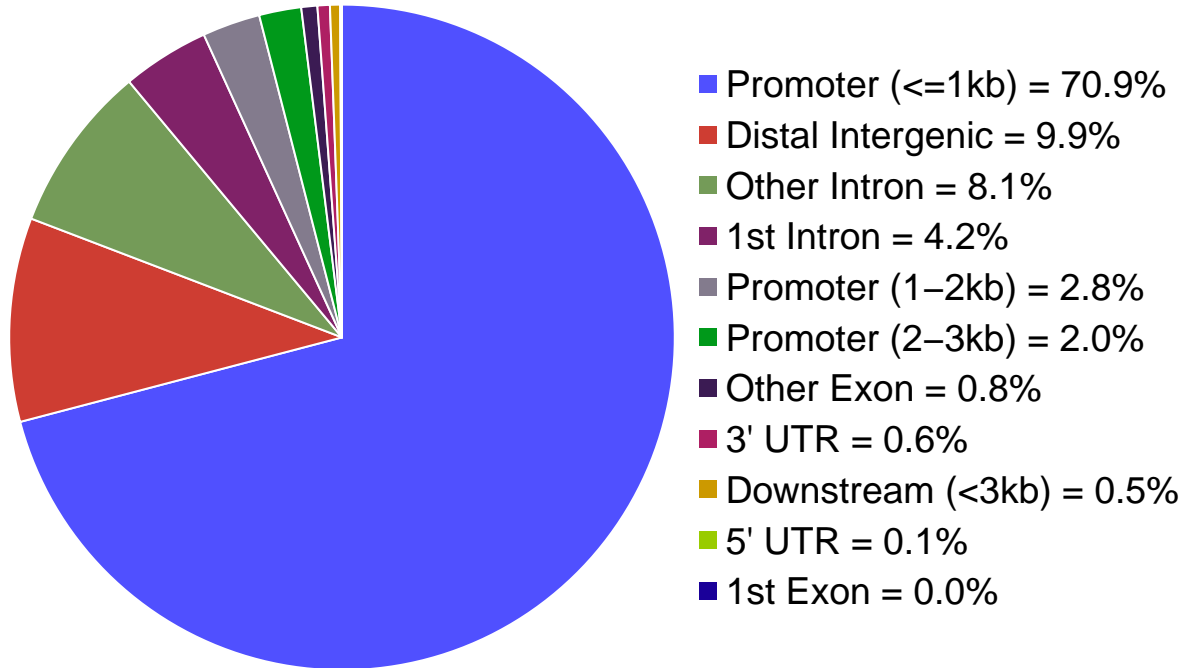

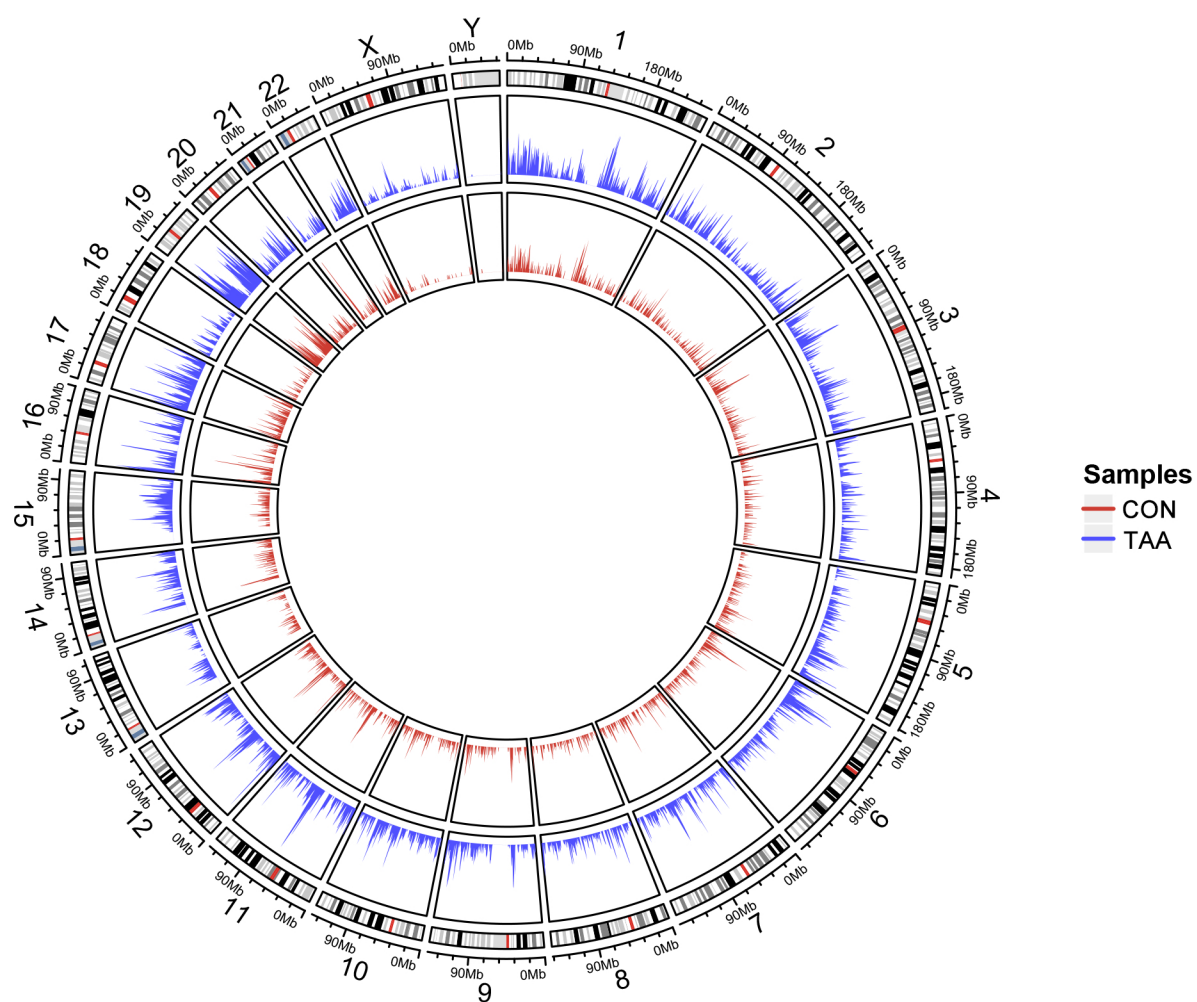

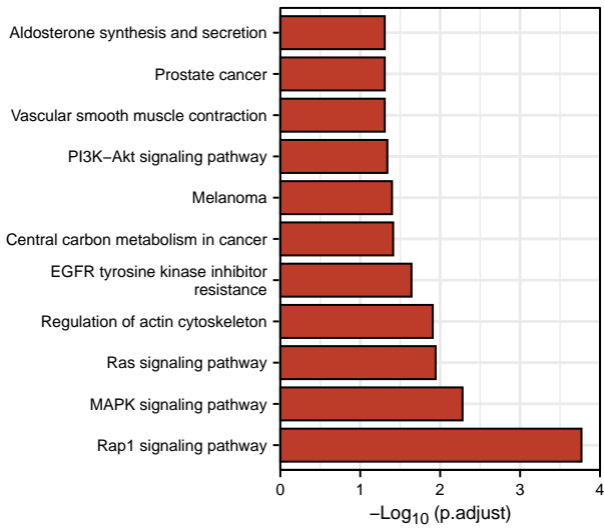

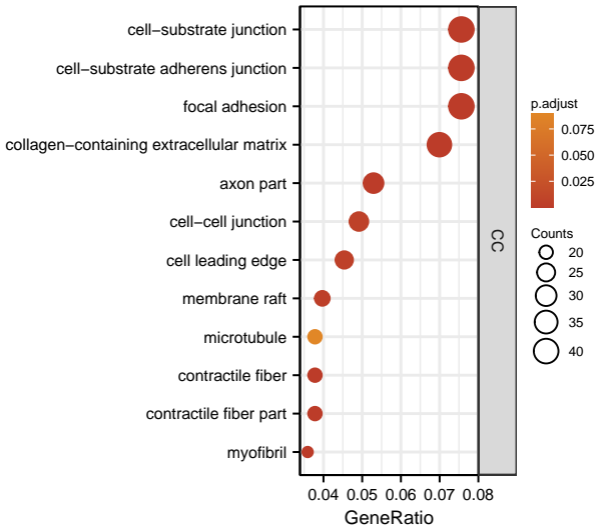

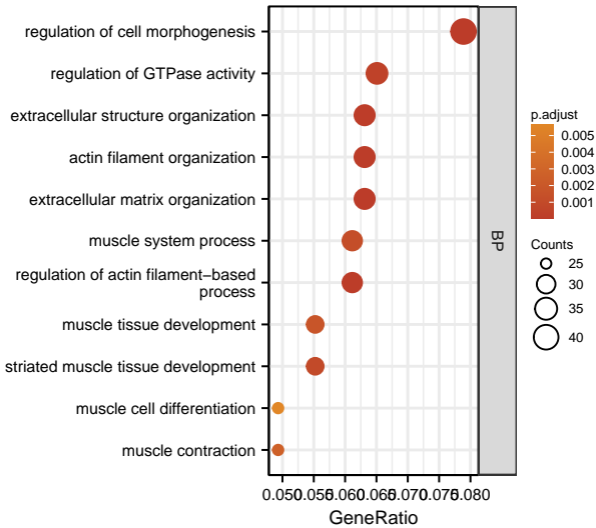

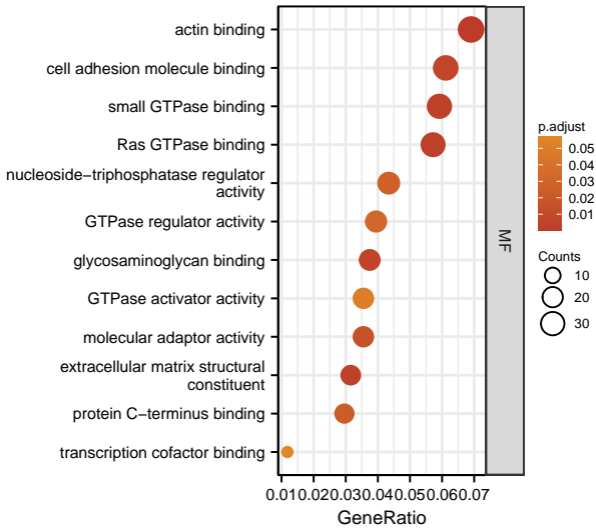

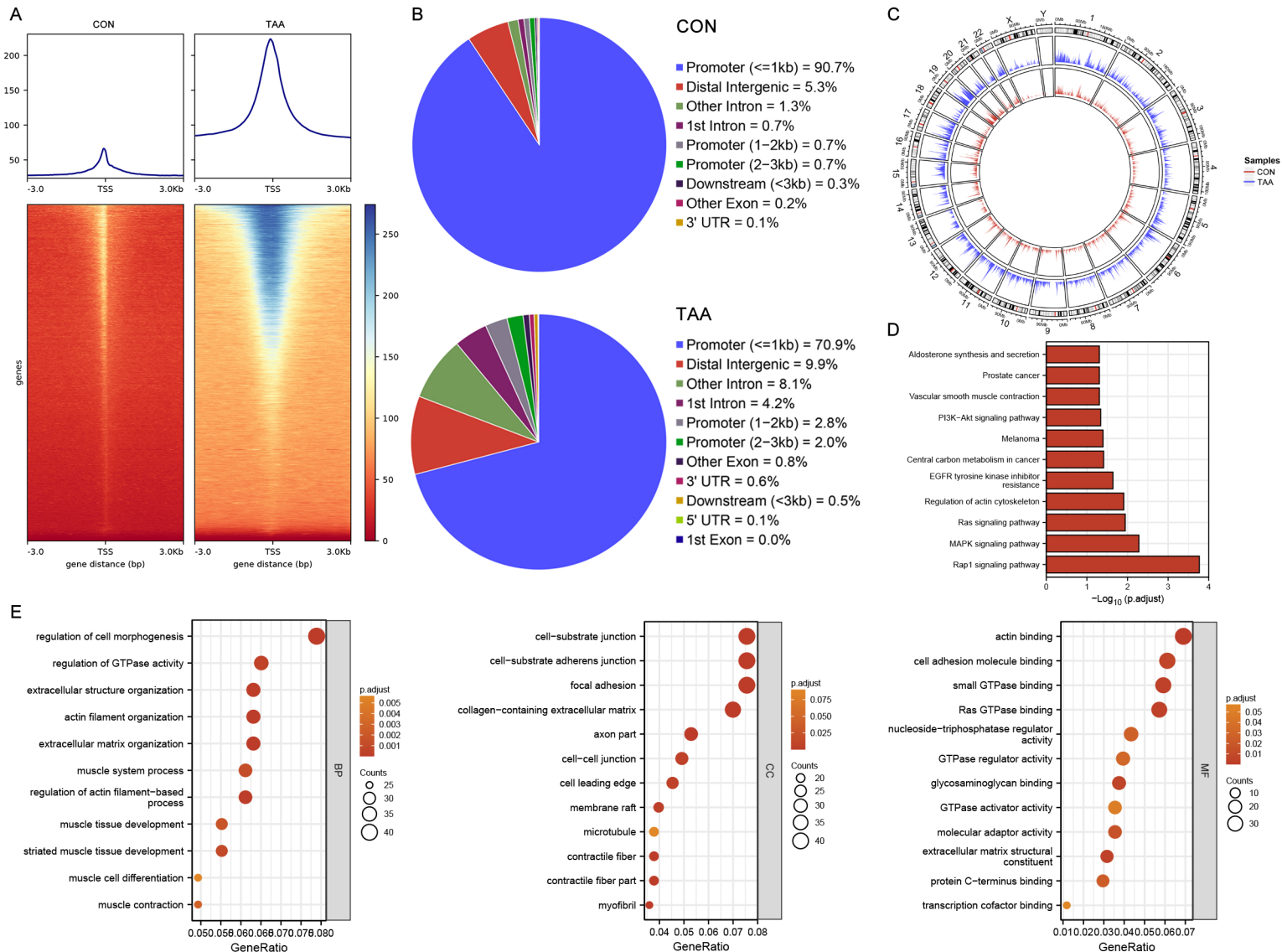

Supplement: Supplementary file 3 [file Datasheet3.pdf]

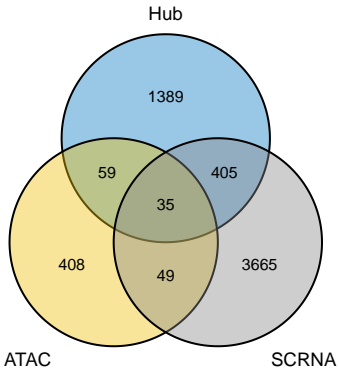

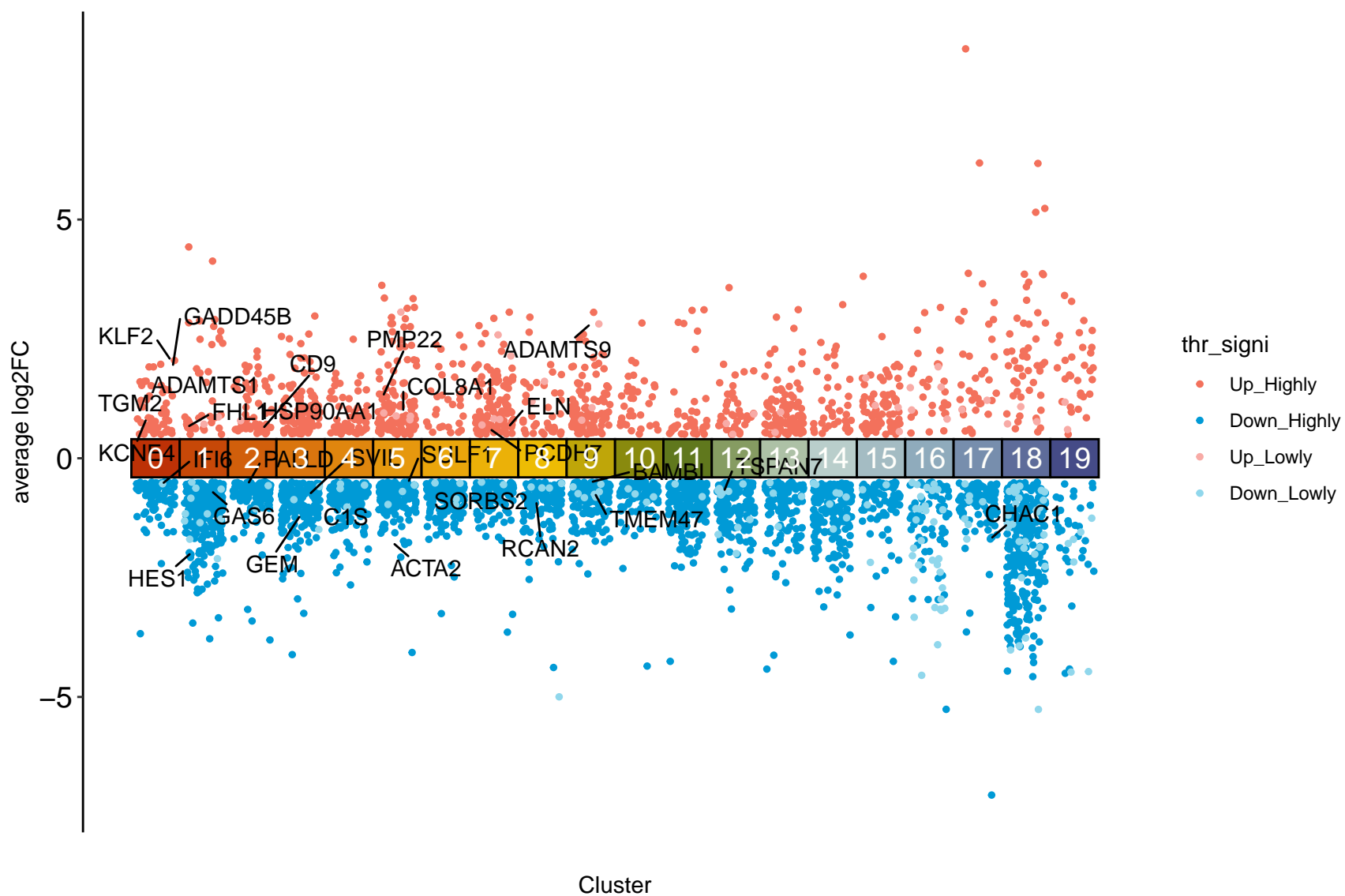

**GAS6**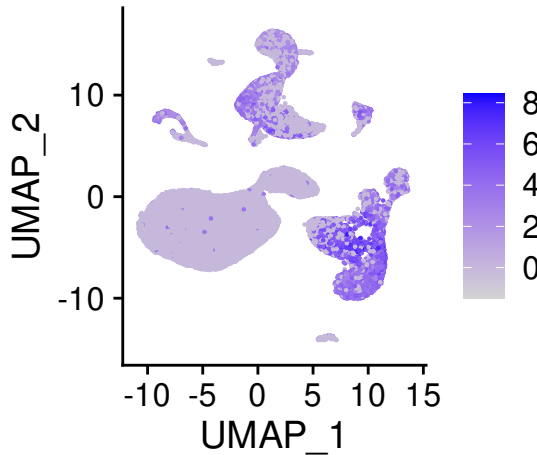**PALLD**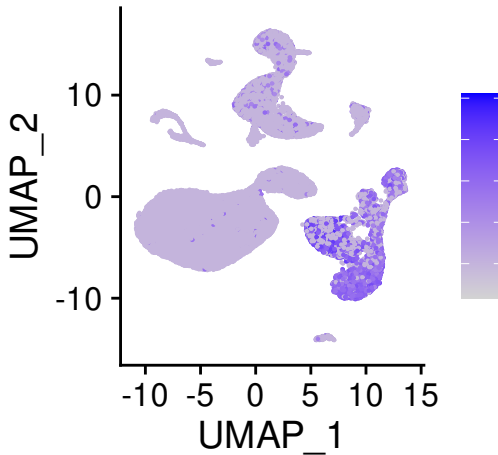**ACTA2**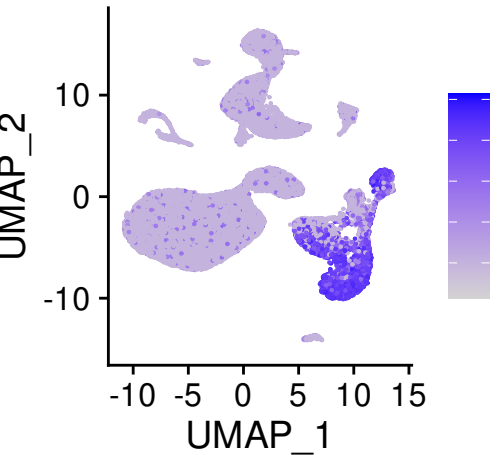**CD9**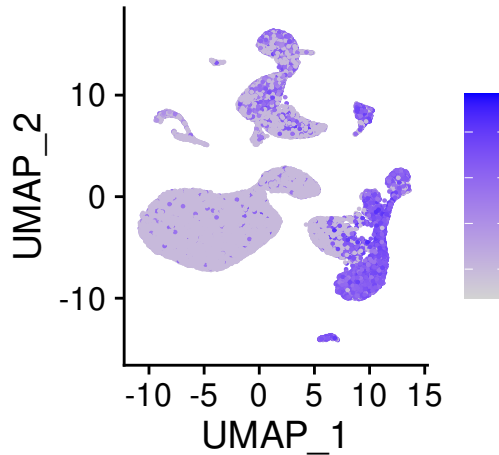**FHL1**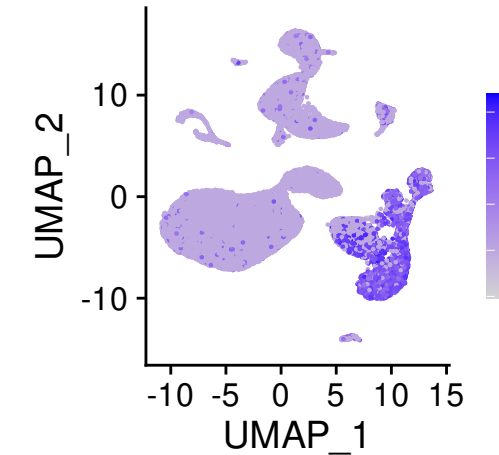**HSP90AA1**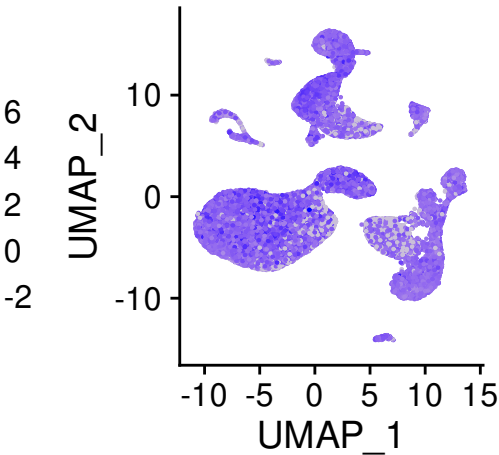

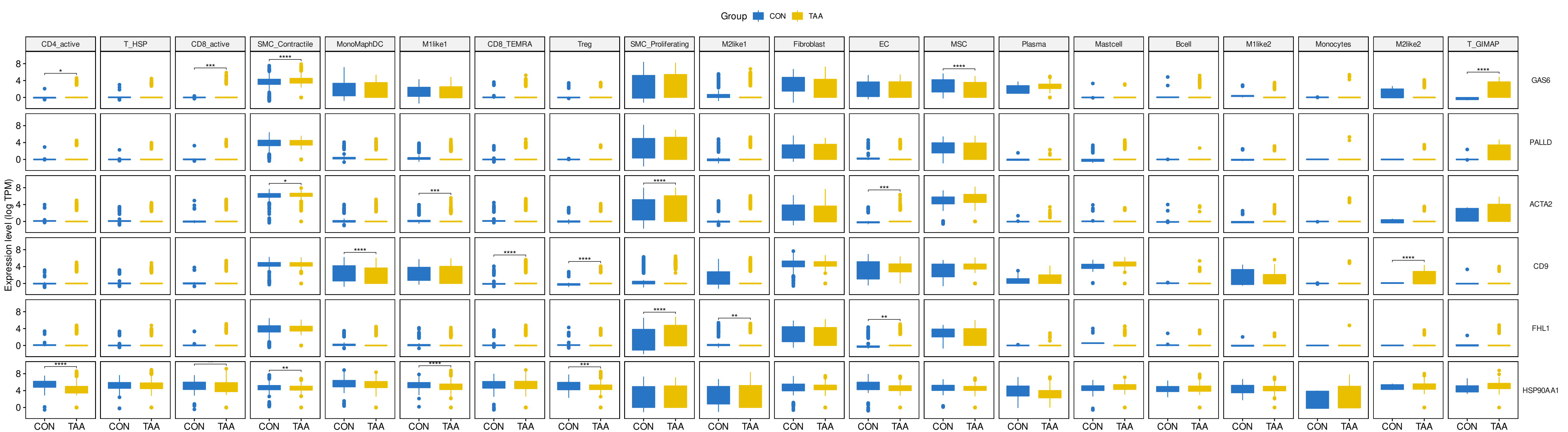

A

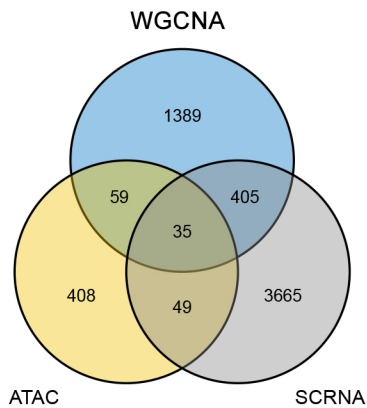

B

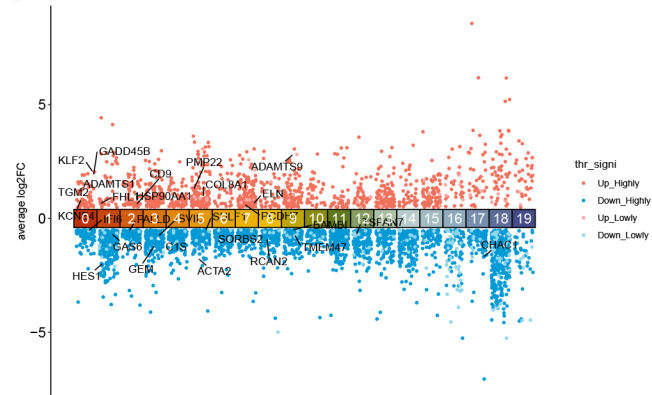

C

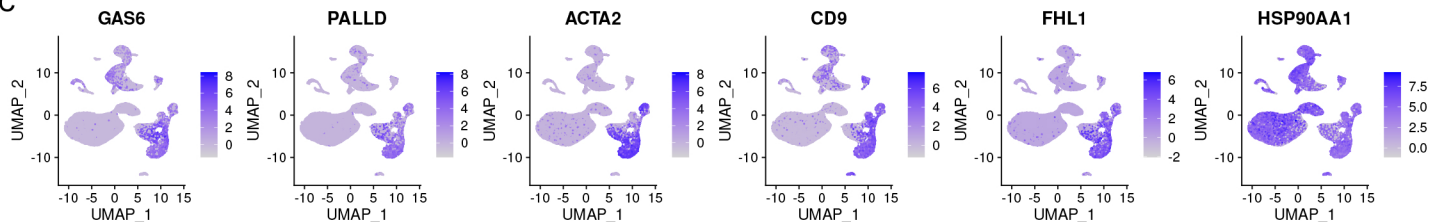

D

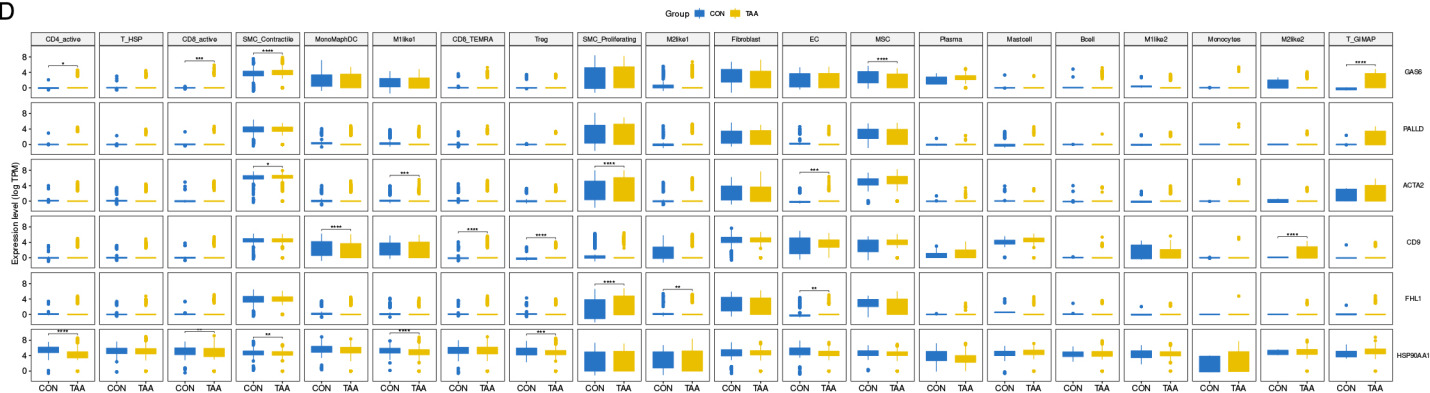

Supplement: Supplementary file 5 [file Datasheet5.pdf]
