# Supplementary material for: Single-cell RNA sequencing and ATAC sequencing identify novel biomarkers for bicuspid aortic valve-associated thoracic aortic aneurysm
Source: Front Cardiovasc Med. 2024 Apr 8;11:1265378. doi: 10.3389/fcvm.2024.1265378 (PMC11057375; doi:10.3389/fcvm.2024.1265378)
Supplement: Supplementary file 4 [file Datasheet4.pdf]

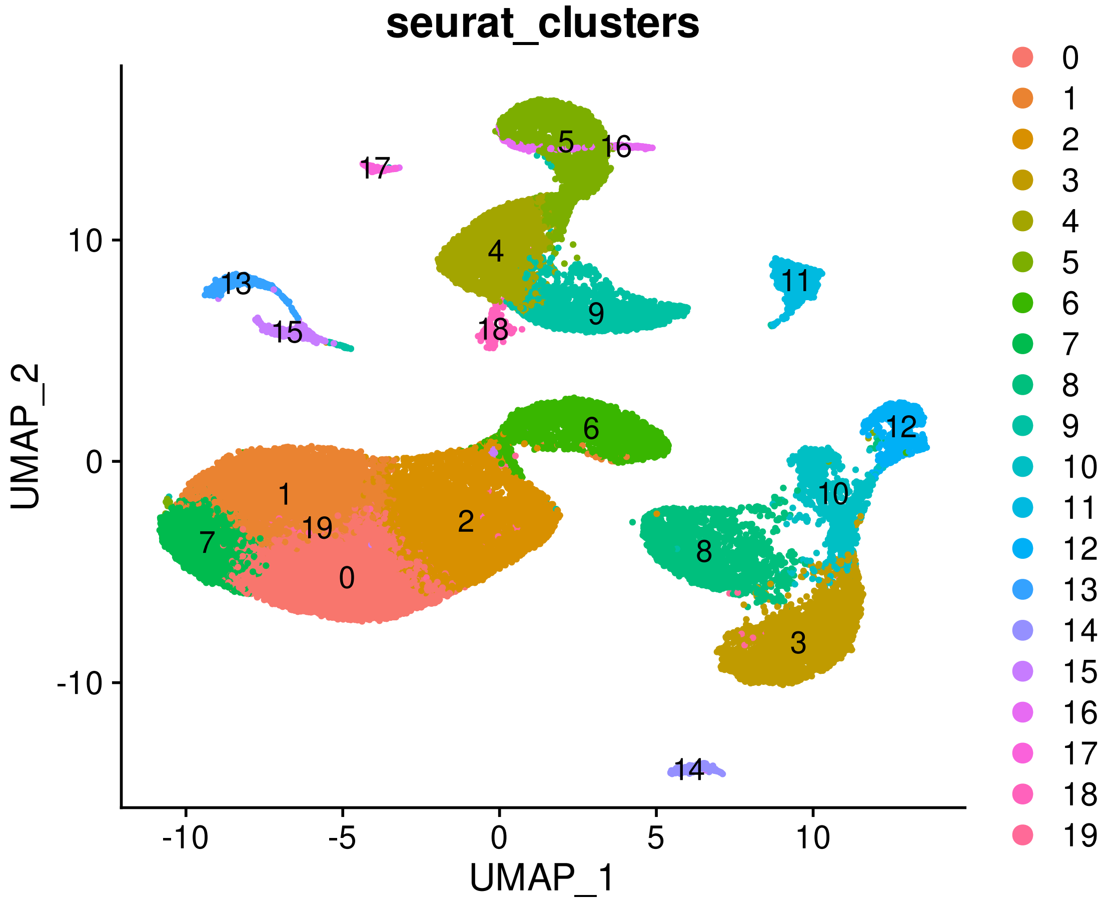
seurat_clusters
UMAP_1
UMAP_2
-10
-5
0
5
10
10
0
-10
0
1
2
3
4
5
6
7
8
9
10
11
12
13
14
15
16
17
18
19

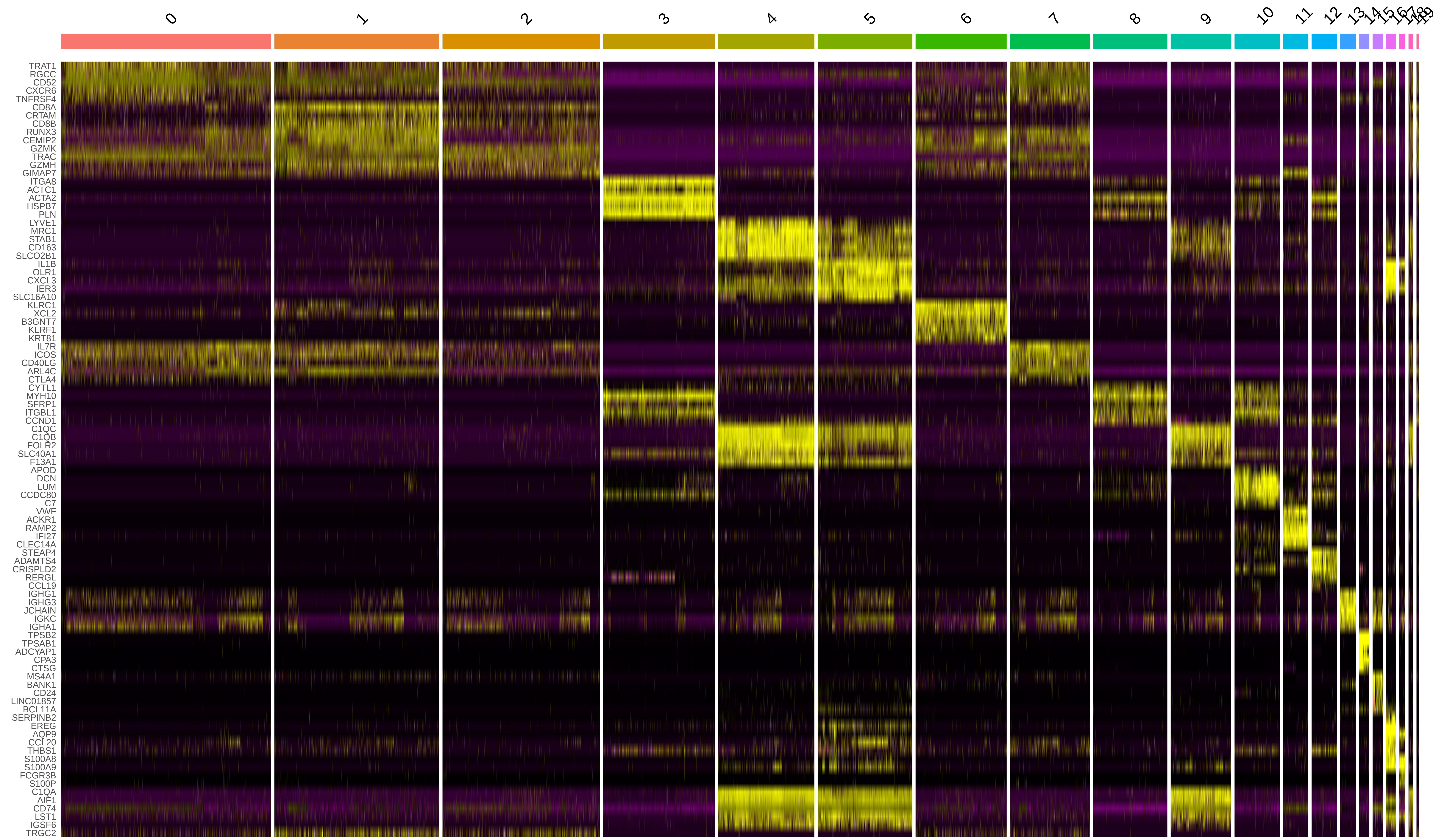
0
1
2
3
4
5
6
7
8
9
10
11
12
13
TRAT1
RGCC
CD52
CXCR6
TNFRSF4
CD8A
CRTAM
CD8B
RUNX3
CEMIP2
GZMK
TRAC
GZMH
GIMAP7
ITGA8
ACTC1
ACTA2
HSPB7
PLN
LYVE1
MRC1
STAB1
CD163
SLCO2B1
IL1B
OLR1
CXCL3
IER3
SLC16A10
KLRC1
XCL2
B3GNT7
KLRF1
KRT81
IL7R
ICOS
CD40LG
ARL4C
CTLA4
CYTL1
MYH10
SFRP1
ITGBL1
CCND1
C1QC
C1QB
FOLR2
SLC40A1
F13A1
APOD
DCN
LUM
CCDC80
C7
VWF
ACKR1
RAMP2
IFI27
CLEC14A
STEAP4
ADAMTS4
CRISPLD2
RERGL
CCL19
IGHG1
IGHG3
JCHAIN
IGKC
IGHA1
TPSB2
TPSAB1
ADCYAP1
CPA3
CTSG
MS4A1
BANK1
CD24
LINC01857
BCL11A
SERPINB2
EREG
AQP9
CCL20
THBS1
S100A8
S100A9
FCGR3B
S100P
C1QA
AIF1
CD74
LST1
IGSF6
TRGC2

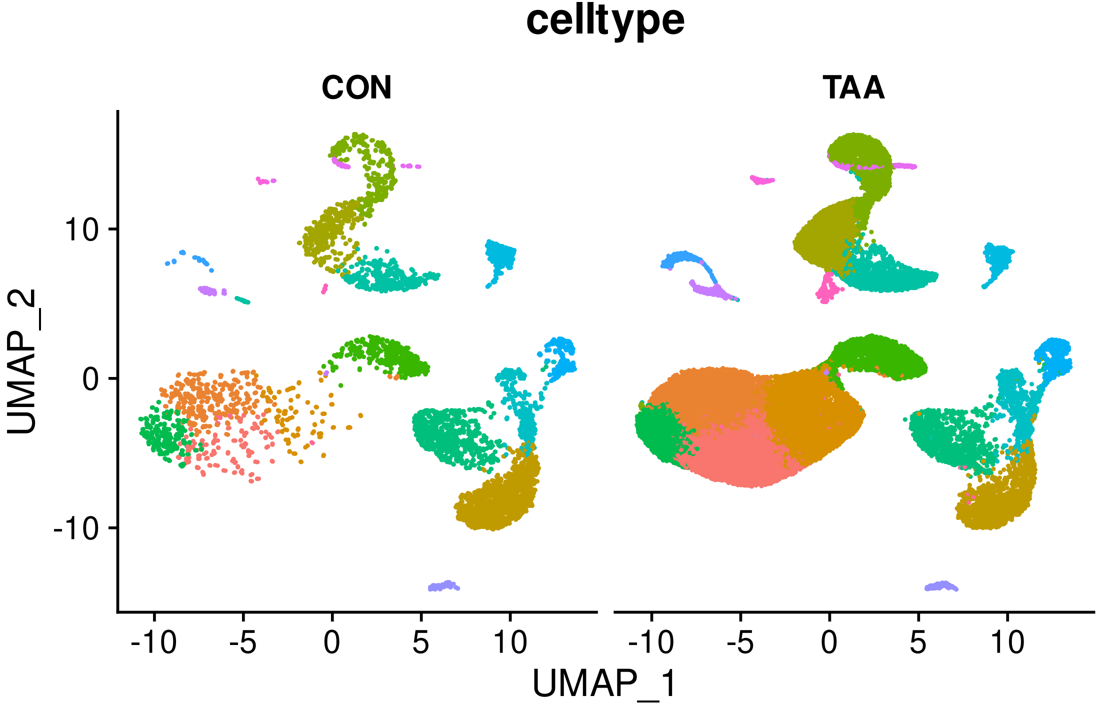
celltype
CON
TAA
UMAP_2
UMAP_1
10
0
-10
-10
-5
0
5
10

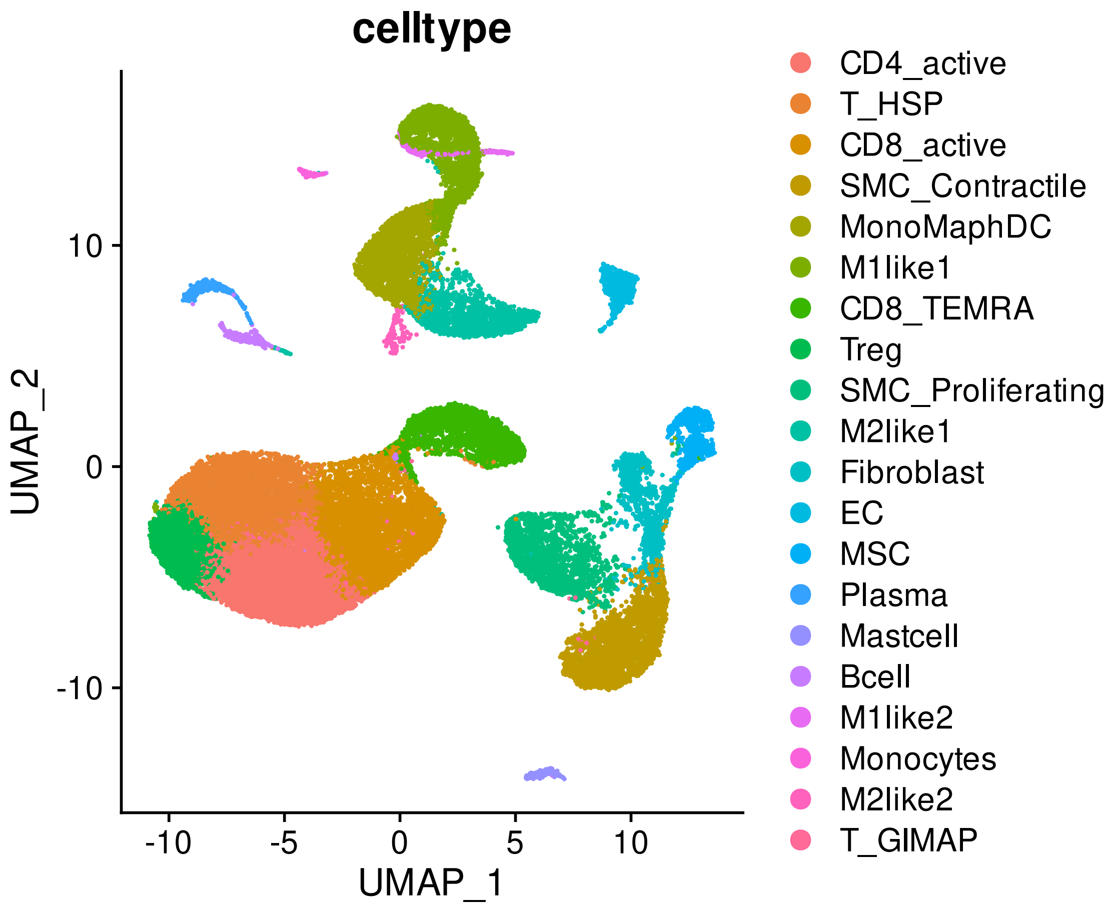
celltype
UMAP_2
UMAP_1
10
0
-10
-10
-5
0
5
10
CD4_active
T_HSP
CD8_active
SMC_Contractile
MonoMaphDC
M1like1
CD8_TEMRA
Treg
SMC_Proliferating
M2like1
Fibroblast
EC
MSC
Plasma
Mastcell
Bcell
M1like2
Monocytes
M2like2
T_GIMAP

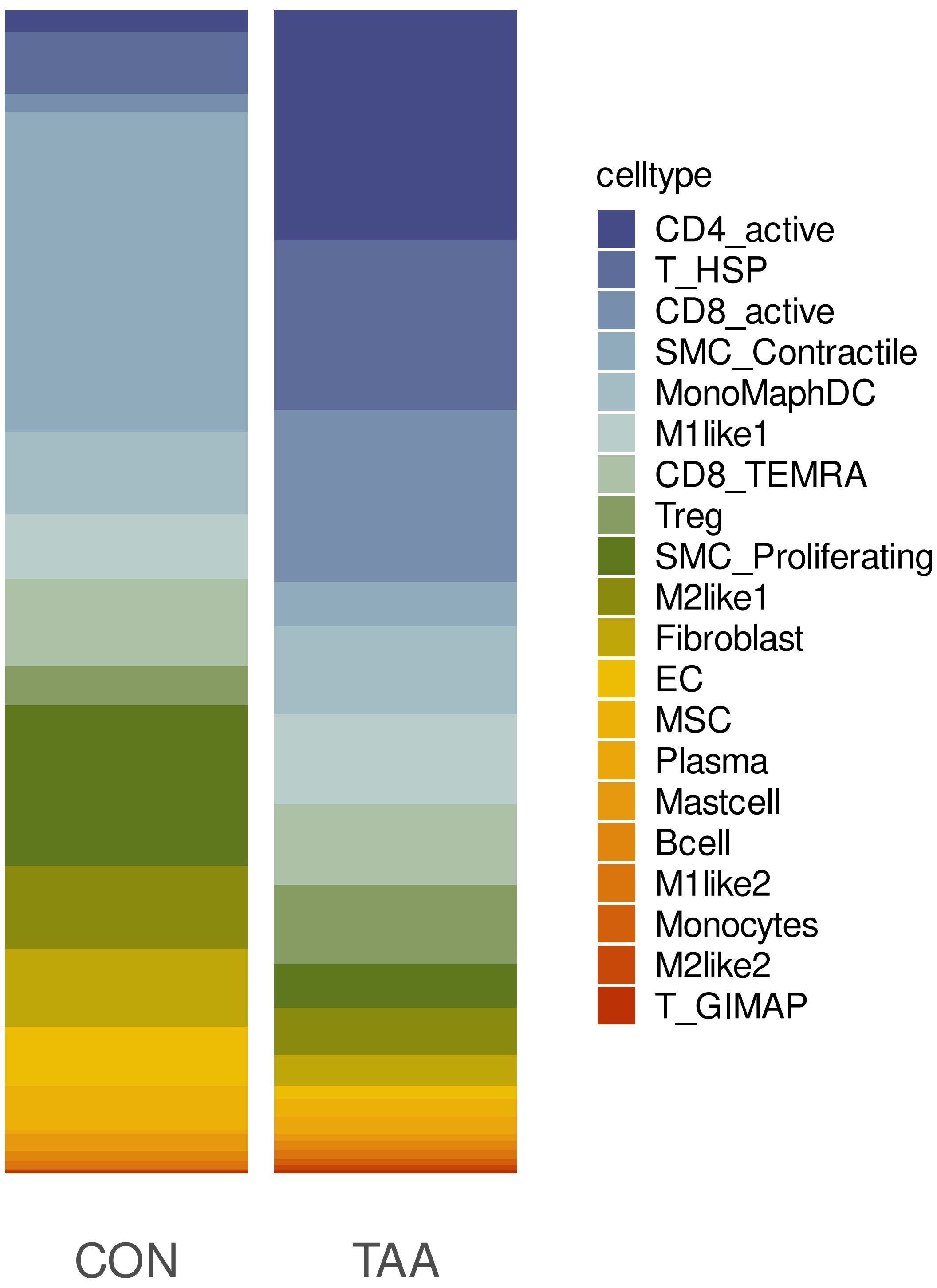
celltype
CD4_active
T_HSP
CD8_active
SMC_Contractile
MonoMaphDC
M1like1
CD8_TEMRA
Treg
SMC_Proliferating
M2like1
Fibroblast
EC
MSC
Plasma
Mastcell
Bcell
M1like2
Monocytes
M2like2
T_GIMAP
CON
TAA

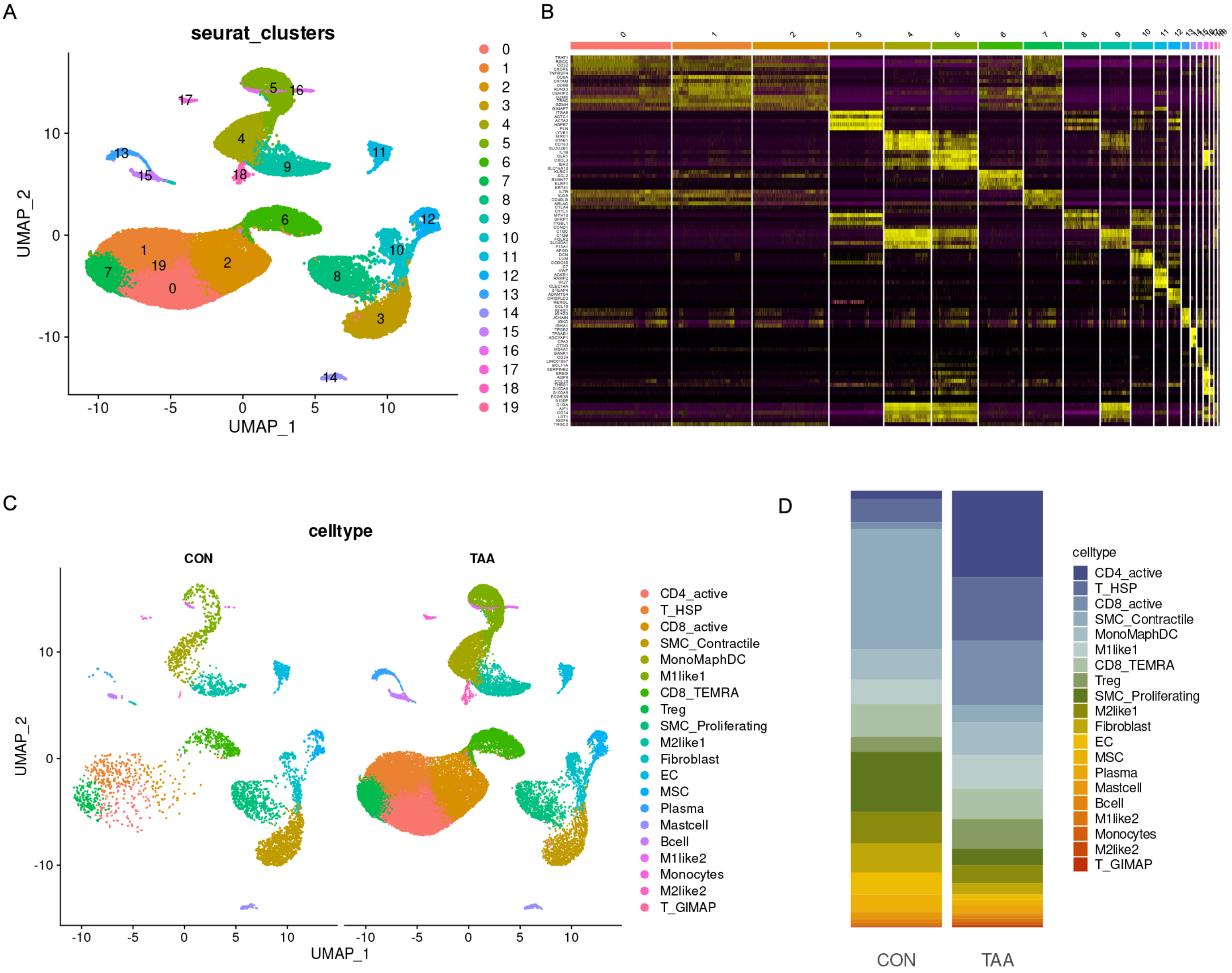
A
seurat_clusters
UMAP_2
UMAP_1
10
0
-10
-10
-5
0
5
10
0
1
2
3
4
5
6
7
8
9
10
11
12
13
14
15
16
17
18
19
B
C
celltype
CON
TAA
UMAP_2
UMAP_1
CD4_active
T_HSP
CD8_active
SMC_Contractile
MonoMaphDC
M1like1
CD8_TEMRA
Treg
SMC_Proliferating
M2like1
Fibroblast
EC
MSC
Plasma
Mastcell
Bcell
M1like2
Monocytes
M2like2
T_GIMAP
D
celltype
CD4_active
T_HSP
CD8_active
SMC_Contractile
MonoMaphDC
M1like1
CD8_TEMRA
Treg
SMC_Proliferating
M2like1
Fibroblast
EC
MSC
Plasma
Mastcell
Bcell
M1like2
Monocytes
M2like2
T_GIMAP
CON
TAA
