# Supplementary material for: Single-cell RNA sequencing and ATAC sequencing identify novel biomarkers for bicuspid aortic valve-associated thoracic aortic aneurysm
Source: Front Cardiovasc Med. 2024 Apr 8;11:1265378. doi: 10.3389/fcvm.2024.1265378 (PMC11057375; doi:10.3389/fcvm.2024.1265378)
Supplement: Supplementary file 6 [file Datasheet6.pdf]

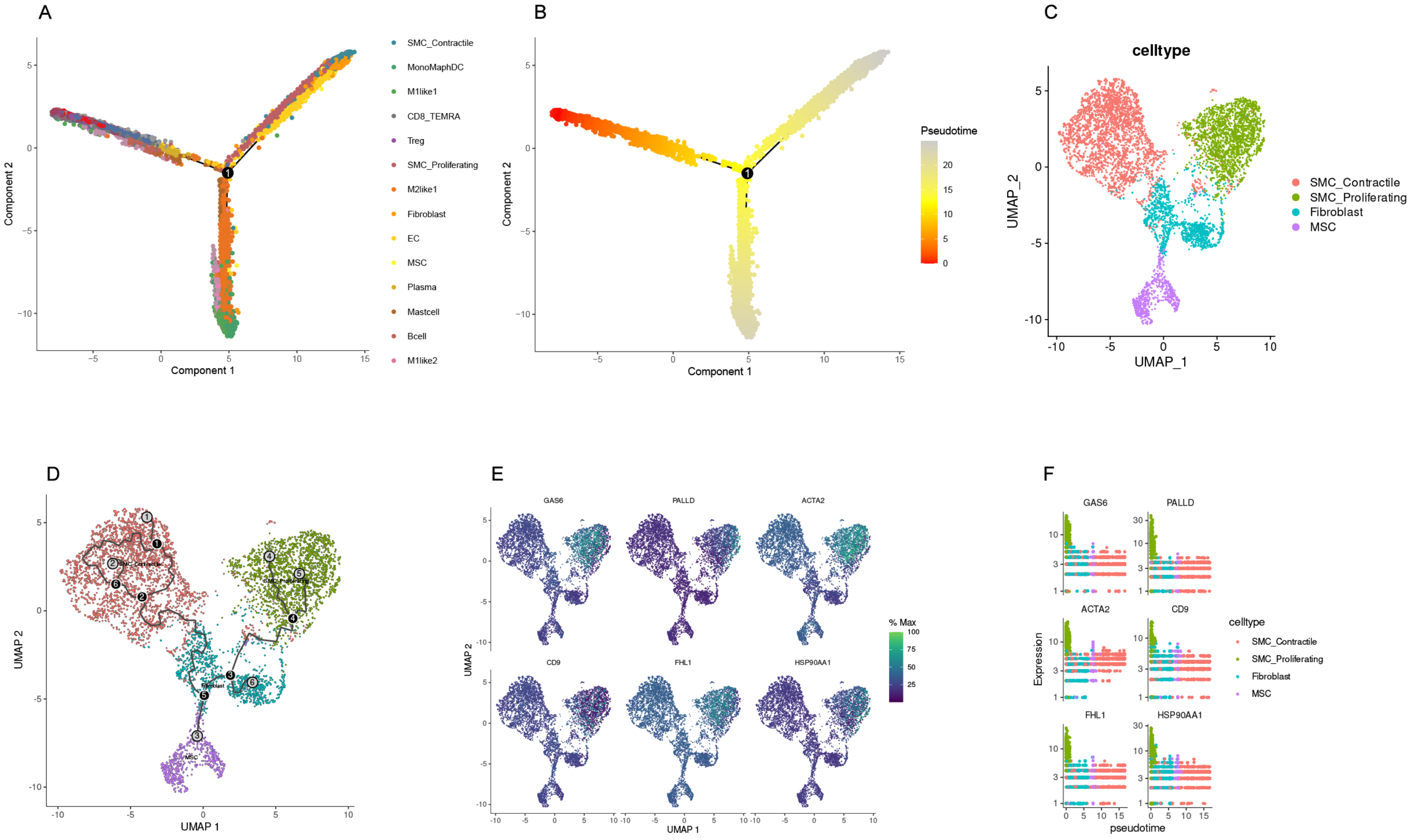
A
B
C
D
E
F
Component 1
Component 2
SMC_Contractile
MonoMaphDC
M1like1
CD8_TEMRA
Treg
SMC_Proliferating
M2like1
Fibroblast
EC
MSC
Plasma
Mastcell
Bcell
M1like2
Pseudotime
celltype
UMAP_1
UMAP_2
SMC_Contractile
SMC_Proliferating
Fibroblast
MSC
UMAP 1
UMAP 2
GAS6
PALLD
ACTA2
CD9
FHL1
HSP90AA1
% Max
Expression
pseudotime

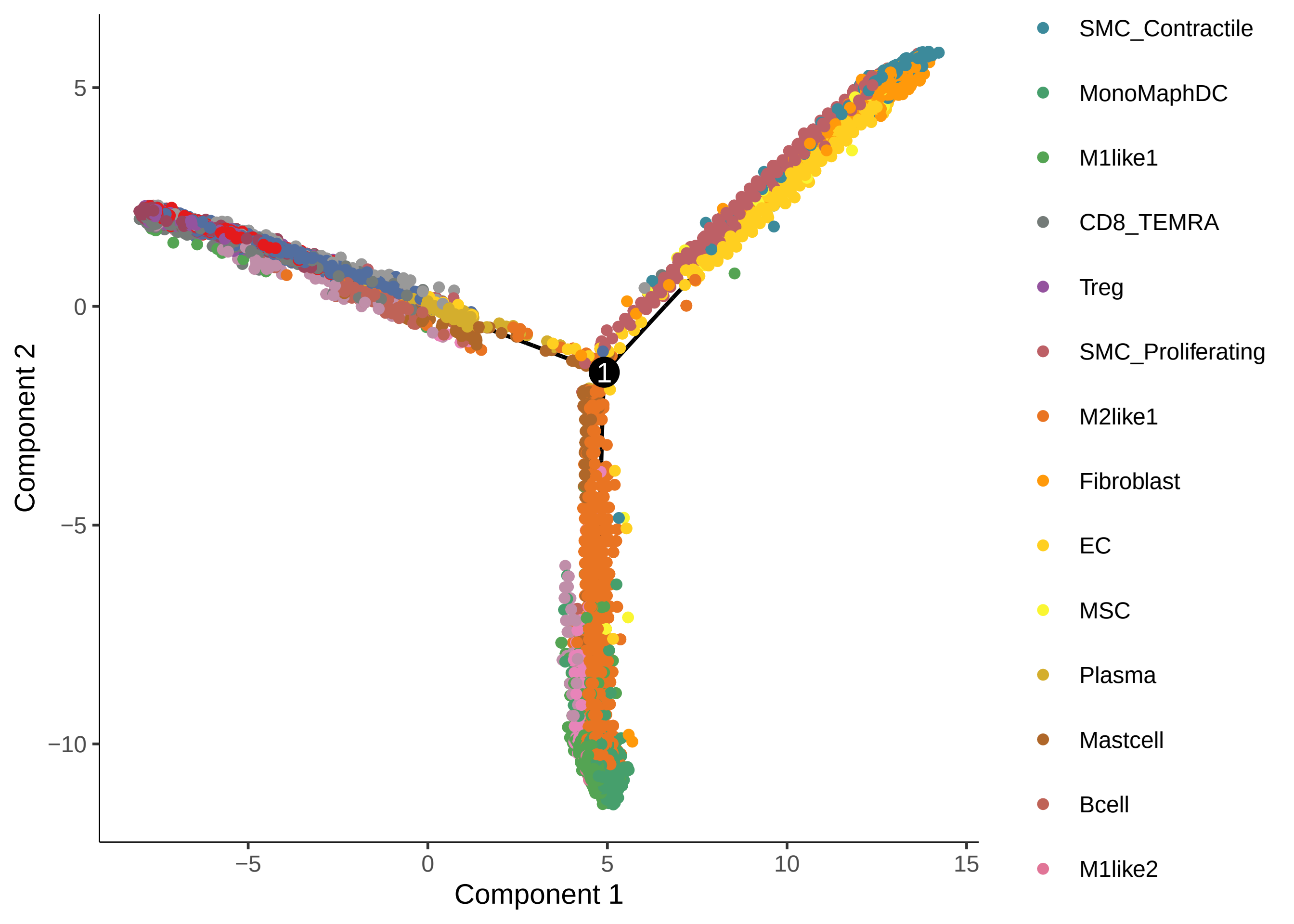

5
0
−5
−10
Component 2
−5
0
5
10
15
Component 1
1
SMC_Contractile
MonoMaphDC
M1like1
CD8_TEMRA
Treg
SMC_Proliferating
M2like1
Fibroblast
EC
MSC
Plasma
Mastcell
Bcell
M1like2

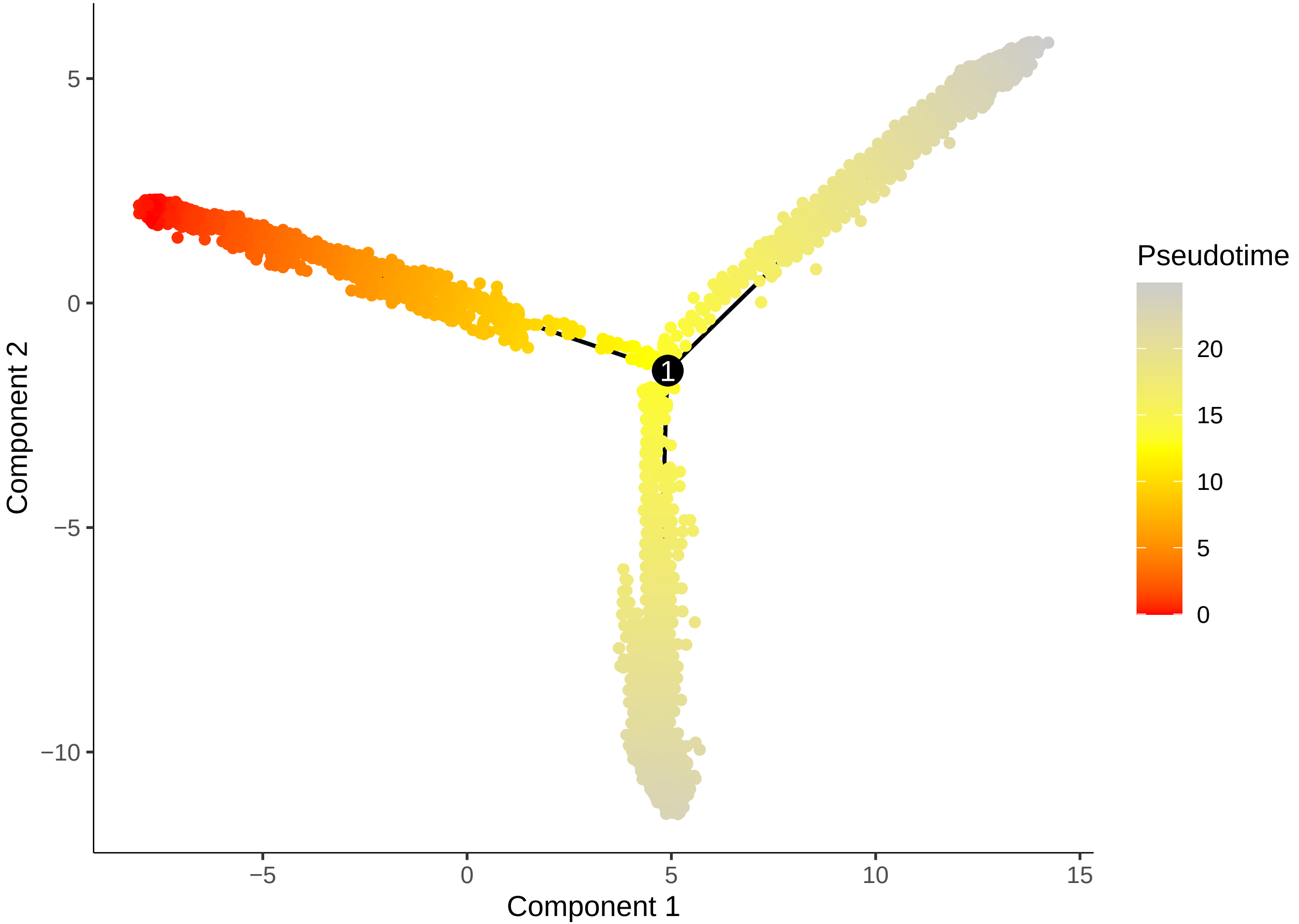

5
0
−5
−10
−5
0
5
10
15
Component 1
Component 2
1
Pseudotime
20
15
10
5
0

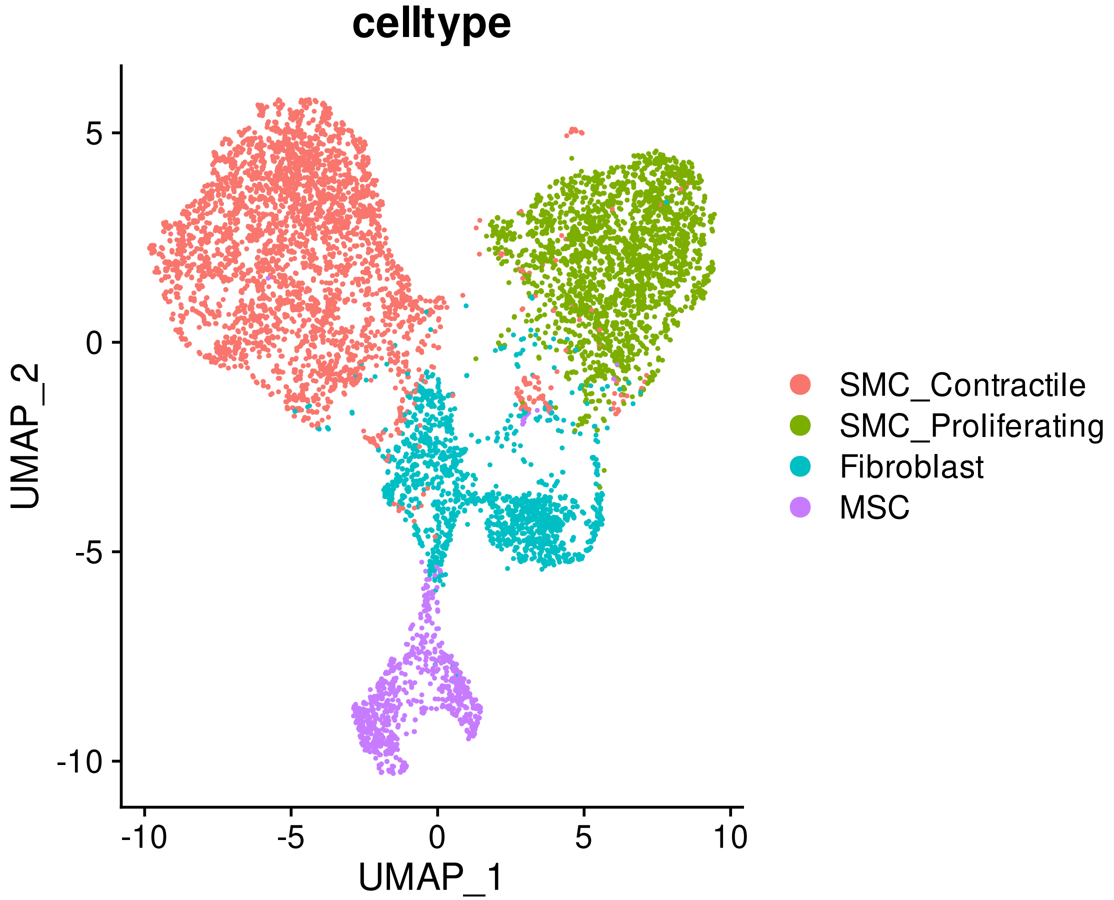
celltype
UMAP_2
UMAP_1
5
0
-5
-10
-10
-5
0
5
10
SMC_Contractile
SMC_Proliferating
Fibroblast
MSC

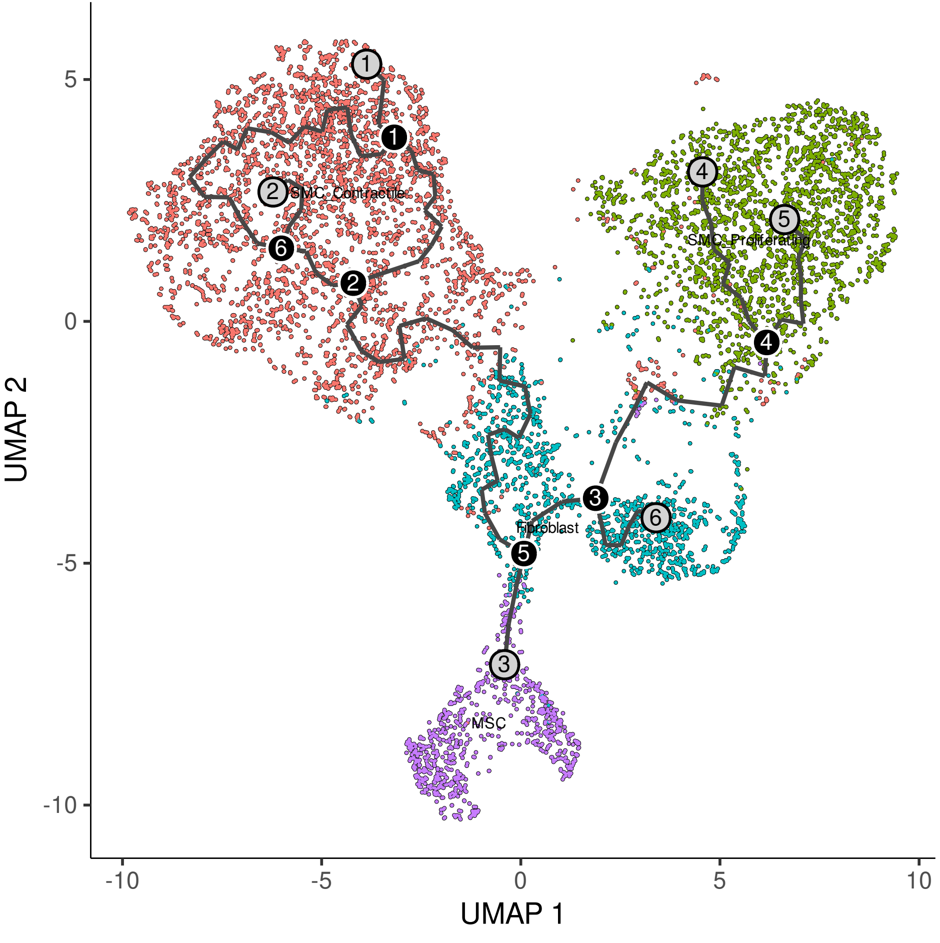

SMC_Contractile
SMC_Proliferating
Fibroblast
MSC
UMAP 1
UMAP 2
5
0
-5
-10
10

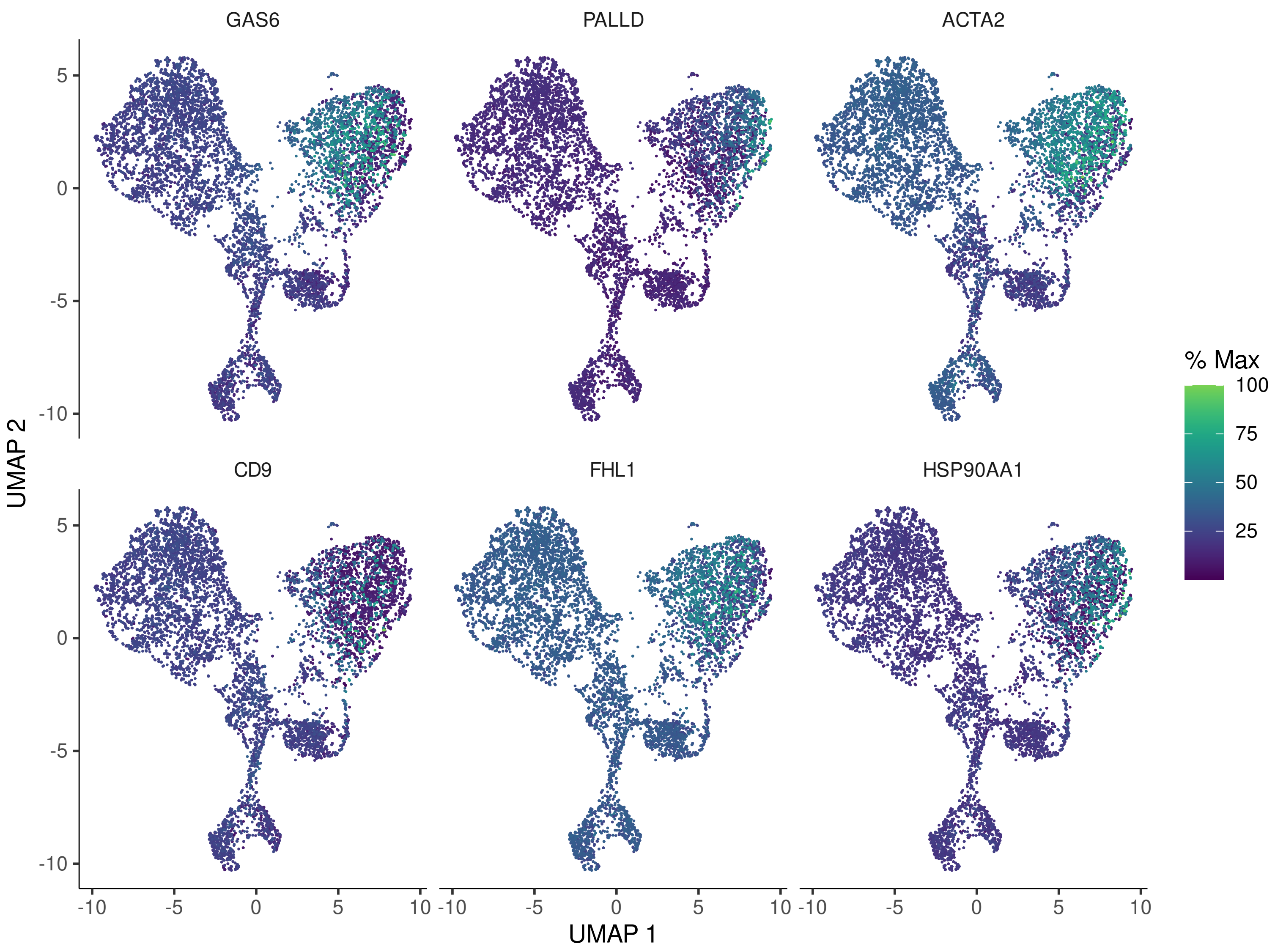
GAS6
PALLD
ACTA2
CD9
FHL1
HSP90AA1
UMAP 2
UMAP 1
% Max
100
75
50
25
5
0
-5
-10
10

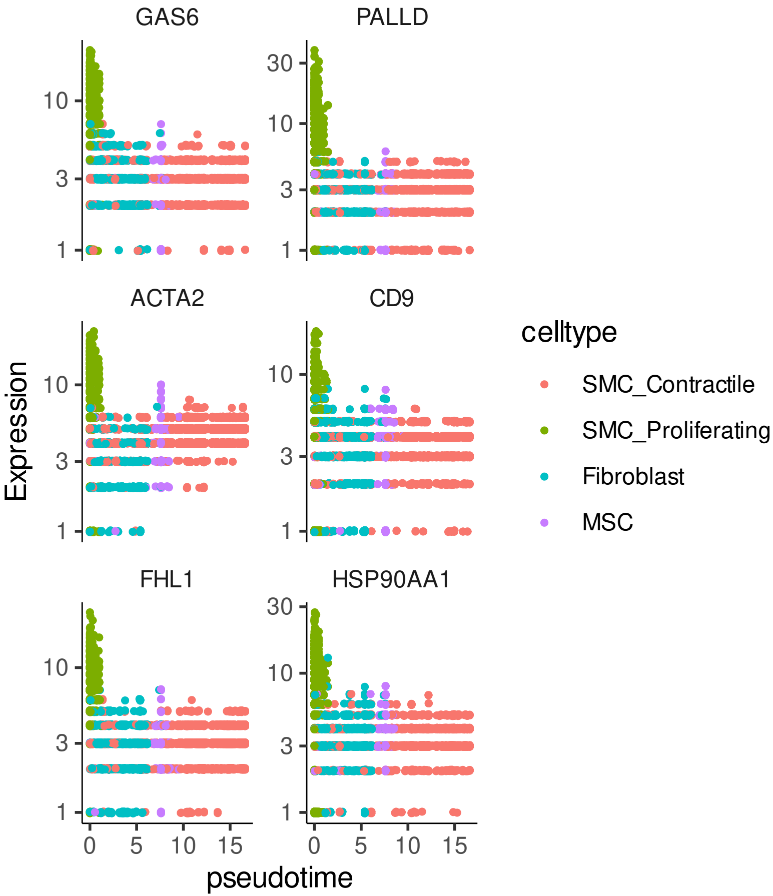

GAS6
PALLD
ACTA2
CD9
FHL1
HSP90AA1
Expression
pseudotime
celltype
SMC_Contractile
SMC_Proliferating
Fibroblast
MSC
